# Advanced Thermoelectric Textiles for Power Generation: Principles, Design, and Manufacturing

**DOI:** 10.1002/gch2.202300023

**Published:** 2023-07-19

**Authors:** Xinyi Chen, Xiaona Yang, Xue Han, Zuping Ruan, Jinchuan Xu, Fuli Huang, Kun Zhang

**Affiliations:** ^1^ Key Laboratory of Textile Science & Technology Ministry of Education Donghua University Shanghai 200051 China; ^2^ College of Textiles Donghua University Shanghai 200051 China

**Keywords:** interfacial interaction, power generation, textile, thermal resistance, thermoelectrics

## Abstract

Self‐powered wearable thermoelectric (TE) devices significantly reduce the inconvenience caused to users, especially in daily use of portable devices and monitoring personal health. The textile‐based TE devices (TETs) exhibit the excellent flexibility, deformability, and light weight, which fulfill demands of long‐term wearing for the human body. In comparison to traditional TE devices with their longstanding research history, TETs are still in an initial stage of growth. In recent years, TETs to provide electricity for low‐power wearable electronics have attracted increasing attention. This review summarizes the recent progress of TETs from the points of selecting TE materials, scalable fabrication methods of TE fibers/yarns and TETs, structure design of TETs and reported high‐performance TETs. The key points to develop TETs with outstanding TE properties and mechanical performance and better than available optimization strategies are discussed. Furthermore, remaining challenges and perspectives of TETs are also proposed to suggest practical applications for heat harvesting from human body.

## Graphical abstract

This review delivers insights into thermoelectric textiles from the aspects of the selection and optimization of thermoelectric materials, their interfacial properties with textile fibers as well as configuration design and scalable fabrication of thermoelectric textiles. Possible rational ways to address the plight of designing and manufacturing high‐performance thermoelectric textiles are proposed.

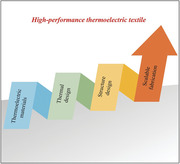

## 1 Introduction

The Internet of Things (IoTs) is a network of interconnected devices, which achieves great success in wearable electronics for healthcare, machine‐learning, social interactions, and entertainment.^[^ ^1^, ^2^, ^3^ ^]^ Relying on the number and size of electronics and complexity of electronic circuits, power requirement ranges from a few microwatts (µW) to watt (W).^[^ ^4^, ^5^ ^]^ The rapid growth in wearable IoTs demands a large‐scale, affordable, and uninterrupted power supply, where sustainable and wearable power generators are specially needed. Except wearable solar cells, the total daily activities of adults can emit heat energy of ≈60–180 W.^[^ ^6^ ^]^ If 1% of the body heat can be harvested and converted to electricity, it may satisfy the requirement of powering many wearable electronics.^[^ ^7^ ^]^ That is, the promising wearable thermoelectric generators (TEGs) with no moving parts and solid‐state conversion show great promise to them, which can directly, ubiquitously, and sustainably convert heat energy from human body to electricity via Seebeck effect. Traditional TEGs, consisting of n‐type and p‐type TE legs connected electrically in series and thermally in parallel, are rigid with inferior flexibility. Nevertheless, for long‐term wearing scenarios, TEGs should possess wearing comfortability and not limit daily movement and metabolism of human body. Some researchers developed flexible TEGs by reducing the size of TEGs or depositing TE materials on flexible substrates, whereas these strategies are limited in designing bendable but not truly wearable TEGs as common clothes.

**Figure 1:**
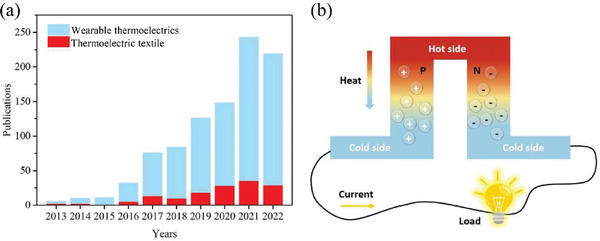
a) Numbers of publications including articles and review papers on the topic of thermoelectric and thermoelectric textiles in recent years. b) The schematic diagram of a TE module on its power generation mode.

Fiber‐based clothes have been developed and used by human beings for more than 10 000 years because of their comfortability and conformability on human body.^[^ ^8^ ^]^ In the past decade, textile‐based TE devices (TETs) have attracted more and more attentions and become one emerging division of wearable TEGs (**Figure** **1**a), though it is still in its initial stage. TETs assembled from fibers, filaments and fabrics constitute the frontier of wearable TEGs that are of 3D comfortability, referring to flexible, stretchable, and twistable, to the dynamic curved surface on human body.^[^ ^9^ ^]^ However, they still face many challenges in materials synthesis, structure design, and scalable manufacture of TETs.

Suitable TE material candidates for TETs require light weight, nontoxicity, chemical and mechanical stability, good flexibility and stretchability, or strong interaction with traditional textile fibers.^[^ ^10^ ^]^ Organic TE materials usually fulfill above requirements whereas their low TE properties still make them rather difficult to address practical issues in daily life. In contrast, majority of inorganic TE materials possesses high TE properties but limited mechanical deformation capability and poor interaction with textile fibers. On the other hand, on account of the thermal mismatching between human body, device, and environment, it is still challenging to build a large temperature difference for high performance in heat‐to‐electricity conversion.^[^ ^11^ ^]^ Though there are some reviews on wearable thermoelectric devices, there are limited TETs with in‐depth but systematic understanding. So it is highly demanded to systematically review the research progress of TETs toward significant mechanical properties and energy conversion efficiency. In contrast to others, this review intends to deliver insights on TETs from the aspects of the selection and optimization of TE materials, their interfacial properties with textile fibers as well as configuration design and scalable fabrication of TETs.

## 2 Basic Knowledge

TETs share the similar fundamental mechanism to that of traditional TE devices. Its thermoelectric power generation is based on the Seebeck effect, which describes the direct conversion between thermal energy to electrical energy by applying a temperature difference on top and bottom sides of devices (Figure 1b). The energy harvesting in TET from body heat is largely affected by the thermal conditions of human body and environment and their thermal interactions with TET. In the following parts, we will give brief introduction to the available body heat energy, and the basic principles, evaluation methods of output, and wearing performance of TET.

### 2.1 Human Heat

**Figure 2:**
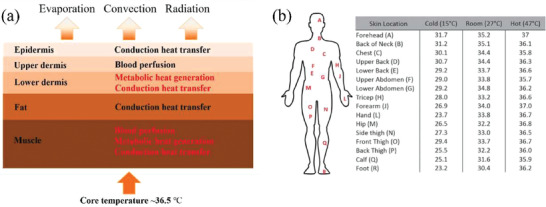
a) Schematic thermal profile of human body. Reproduced with permission.^[^ ^13^ ^]^ Copyright 2019, Elsevier. b) The local skin temperatures at different body locations at various ambient temperatures. Reproduced with permission.^[^ ^16^ ^]^ Copyright 1992, Springer Nature. Reproduced with permission.^[^ ^17^ ^]^ Copyright 2016, Royal Society of Chemistry.

Among the generated energy by metabolism in human body, only ≈15–30% of energy is converted in to the form of mechanical energy while a large amount of that in form of heat energy is used for keeping constant body temperature or dissipated as waste heat.^[^ ^6^ ^]^ Therefore, the homothermal human body can be regarded as a sustainable heat source with a constant core temperature of ≈36.5 °C.^[^ ^12^ ^]^ The heat dissipation from body skin includes thermal conduction, radiation, convection, and sweat evaporation (**Figure** **2**a).^[^ ^13^ ^]^ The heat dissipations from metabolism are forced heat and mass transfer in lungs and the heat loss by excretion which is usually neglected in calculation.^[^ ^14^ ^]^ Since TETs directly contact with body skin, the heat dissipation from skin should be mainly considered for TET.^[^ ^14^, ^15^ ^]^

The average heat density on body skin is ≈7 mW cm^−2^ for a person with sedentary activity in office.^[^ ^14^ ^]^ It can range from 38 to 44 mW cm^−2^ when people conduct moderate or strenuous exercises. It is worthy to noting that the skin temperature is stable at ≈32–35 °C at room temperature and thus the enhancement of heat flow from skin can be neglected. Actually, additional heat energy aroused by exercises is mainly lost via sweat evaporation, which is a kind of latent heat, thus is difficult to be harvested in TETs.^[^ ^18^ ^]^ Hence, the average heat density on skin for a person resting in thermal comfort condition can be used as a reference. By subtracting the heat transfer via lung and sweat evaporation through skin,^[^ ^19^ ^]^ the available heat density for TETs is ≈5 mW cm^−2^.^[^ ^14^ ^]^

Though human body is homothermal, the skin temperature is location‐dependent and can be affected by environmental conditions including temperature, humidity, wind speed, etc.^[^ ^20^ ^]^ Figure 2b shows the local skin temperatures on human body at different ambient temperatures.^[^ ^16^ ^]^ The skin temperature is higher where blood vessels are neat to skin surface owing to the heat transfer by vasodilation and vasoconstriction.^[^ ^21^ ^]^ For example, the heat flow on wrist with abundant blood vessels (10–20 mW cm^−2^) is higher than the average heat flow of skin (1–10 mW cm^−2^) at ambient temperature of 22 °C.^[^ ^22^ ^]^ Additionally, the local thermal resistance of body surface is another important factor that should be taken into account for TETs.

### 2.2 Output Performance

#### 2.2.1 Thermoelectric Figure of Merit

The thermoelectric conversion efficiency of thermoelectric devices highly depends on the thermoelectric properties of TE materials. The overall thermoelectric property is usually evaluated with a dimensionless thermoelectric figure of merit (*zT*)^[^ ^23^ ^]^

(1) $$zT=\frac{S^{2}\sigma T}{k}$$

where *σ* is the electrical conductivity, *S* is the Seebeck coefficient, *k* is the total thermal conductivity, and *T* is the absolute temperature. Meanwhile, *k* and *σ* of TE materials can be calculated by

(2) $$k=k_{e}+k_{l}$$

(3) $$\sigma =nq\mu_{H}$$

where *k*_e_ and *k*_l_ are the electrical and lattice thermal conductivity, respectively. *n*, *q*, *µ*_H_ are the carrier concentration, charge, and carrier mobility, respectively. For degenerated semiconductors and metals, *S* can be expressed as^[^ ^24^ ^]^

(4) $$S=Tm^{*}\frac{8\pi^{2}k_{B}^{2}}{3eh^{2}}{\left(\frac{\pi }{3n}\right)}^{2/3}$$

where m^*^, *k*_B_, and *h* are the carrier effective mass, Boltzmann constant, and Planck's constant, respectively. Ideal TE materials should possess high *S* and *σ* but low *k*. Nevertheless, *S*, *σ*, and *k* are not independent, which are correlated with carrier concentration, carrier mobility, and band structure.^[^ ^25^ ^]^ For the majority of TE materials, *σ* and *k* are positively correlated whereas *S* and *σ* are inversely correlated. The maximum *zT* can be achieved by the trade‐off between *S*, *σ*, and *k*.^[^ ^26^ ^]^

#### 2.2.2 Open‐Circuit Voltage

The open‐circuit voltage (*V*_TET_) at given temperature difference (Δ*T*) can be calculated by

(5) $$V=N(S_{n}+S_{p})\Delta T$$

where *S*_n_ and *S*_p_ are the absolute values of Seebeck coefficients of n‐type and p‐type TE legs, respectively. *N* is the number of thermocouples.

#### 2.2.3 Output Power and Power Density

The out power and energy conversion efficiency are significant indexes for the device performance.^[^ ^17^ ^]^ When TET is connected to an external electrical resistance load, the output power (*P*) is defined as

(6) $$P=\frac{V^{2}}{{\left(R_{\mathrm{TET}}+R_{\mathrm{load}}\right)}^{2}}\;R_{\mathrm{load}}$$

where *R*_TET_ and *R*_load_ are the internal electrical resistance of TET and the external electrical resistance of load, separately. At a given temperature difference, the maximum output power (*P*_max_) can be achieved as electrical matching reaches: *R*_TEG_ = *R*_load_. However, for practical applications, the thermal resistance matching is more important which will be carefully discussed during the section for TET design. The output power density (*P*_d_) can be calculated by the obtained output power versus the whole surface area of TET

(7) $$P_{d}=\frac{P}{L\times W}$$

where *L* and *W* are the length and width of TET, respectively.

#### 2.2.4 Energy Conversion Efficiency

Carnot efficiency (*η*_c_) denotes an upper limit of heat energy conversion, which can be given by

(8) $$\eta_{c}=\frac{T_{H}-T_{C}}{T_{C}}$$

where *T*_H_ is the core temperature of human body and *T*_C_ is the environment temperature. Based on the Carnot efficiency, the maximum efficiency of TET is 1.6–6.6% for temperature differences of 5–20 °C, whereas the actual efficiency of TEGs is only 0.2–0.8%, far less than Carnot efficiency.^[^ ^11^ ^]^ The maximum conversion efficiency (*η*_max_) of TETs can be expressed as^[^ ^27^ ^]^

(9) $$\eta_{\max}=\frac{T_{H}-T_{C}}{T_{H}}\;\frac{\left[\sqrt{1+ZT}-1\right]}{\left[\sqrt{1+ZT}+\frac{T_{C}}{T_{H}}\right]}$$

where *zT* is the average figure of merit of device, which is usually lower than *zT* of TE materials due to the parasitic effect. The term $\left[\sqrt{1+ZT}-1\right]/\left[\sqrt{1+ZT}+T_{C}/T_{H}\right]$ denotes the heat loss in Joule heating, heat evaporation, and other irreversible heat loss, which may explain why the conversion efficiency of TETs is far below Carnot efficiency.^[^ ^28^ ^]^

### 2.3 Wearability

In addition to output performance, wearing comfort is essential to ensure that TETs can be used to unremittingly harvest heat energy from human body in real wearing scenarios.^[^ ^29^, ^30^ ^]^ Wearing performance of TET involves the biocompatibility (nontoxic and low density), deformability or conformability (high flexibility, stretchability, twistability), durability, and thermal‐wet comfortability (air and moisture permeability).^[^ ^31^ ^]^

In practical applications, the conformability of TETs can influence not only wearing comfort but also energy conversion efficiency because it ensures TETs to be adhered closely to human body with dynamic curved surface. For wearable electronics, flexibility describes that electronics can be easily bent without fracture or plastic deformation, aka in the elastic range.^[^ ^32^ ^]^ Peng and Snyder. developed a formulation to determine the flexibility of materials,^[^ ^33^ ^]^ whereas there is still lack of specific index to evaluate the flexibility of wearable electronics. At present, most of attempts usually use bending radius or bending angle to exhibit the flexibility of devices.^[^ ^34^ ^]^ In contrast, TET is a kind of textiles, it can be measured via the evaluation methods of traditional fabrics. The deformability of fabrics is dependent on the bending stiffness, shear stiffness, and specific weight.^[^ ^35^ ^]^ The bending rigidity of fabrics can be indirectly measured via the weighted‐ring, ring‐loop, or heart‐loop methods because the accurate calculation of bending stiffness is rather difficult for fabrics.^[^ ^36^ ^]^ The stiffness of fabrics also can be estimated by bending length, which is defined as the length of fabric that will be bent depending on its own weight to a certain extent.^[^ ^37^ ^]^ When involving deformation in multiple directions, the shear stiffness of fabrics should be taken into account.^[^ ^35^ ^]^ The shear response of fabrics can be measured by the bias extension test, picture frame test, KES‐F test, and cylinder shear devices.^[^ ^38^ ^]^ The stretchability of TETs generally evaluated by elongation (*ε* =(*l*_1_‐*l*_0_)/*l*_0_), where *l*_0_ is the length of TETs after loading and *l*_1_ is the length of TETs before loading.

Durability defines the duration and structural stability of TETs, mainly involving the chemical resistance, wearing resistance, impact resistance, and fatigue resistance, which is indispensable for TETs applications in daily life. It depends on the stability of fabric integrity, chemical and mechanical properties of TE yarns/fibers, and interaction between TE materials and textile materials. Lastly, the thermal‐wet comfort can be characterized by the common evaluation indexes including the thermal resistance, thermal conductivity, thermal insulation of clothes (CLO), moisture permeability, and water retention rate by using traditional fabric measurements.

## 3 Candidate TE Materials

TETs generally have pairs of p‐type and n‐type TE legs that are alternatively connected electrically in series and thermally in parallel, in order to obtain sufficient output voltage and power. The type of TE materials with good TE properties, mechanical flexibility, and adhesion to textile materials is the cornerstone of design and fabrication of TETs. In general, TE materials can be divided into two categories of organics and inorganics. Organic thermoelectric materials are aimed to simultaneously achieve high TE performance, light density, and good processability for wearable applications.^[^ ^39^ ^]^ There are some other potential organic TE materials, including newly synthesized semiconducting polymers and small conjugated organic molecules used in other fields, such as optoelectronics, field‐effect transistors, etc., with high carrier mobility and Seebeck coefficient, as well as traditional easy‐processable conducting polymers. In contrast, most of inorganic TE materials have been developed for almost a century, aiming to obtain ultrahigh TE performance for their industrial and deep‐space applications, whereas they are not directly suitable for TETs.^[^ ^8^ ^]^

**Figure 3:**
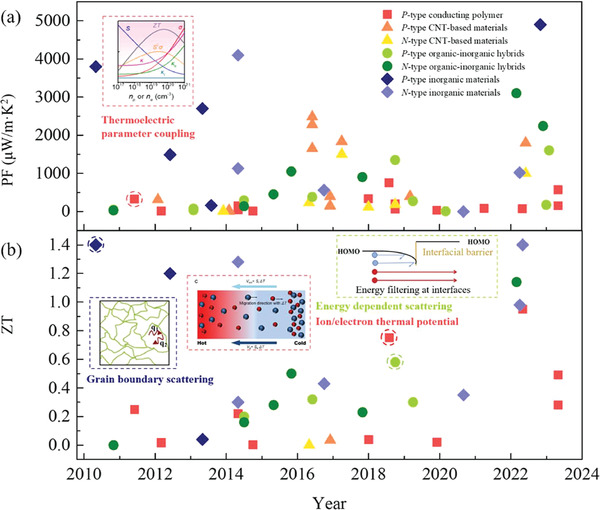
A summary of TE properties of TE materials near room temperature and some underneath working principles underneath TE enhancement^[^ ^23^, ^40^, ^41^ ^]^ over the past decade: a) power factor (PF) and b) thermoelectric figure of merit (*zT*).^[^ ^34^, ^41^, ^42^, ^43^, ^44^, ^45^, ^46^, ^47^, ^48^, ^49^, ^50^, ^51^, ^52^, ^53^, ^54^, ^55^, ^56^, ^57^, ^58^, ^59^, ^60^, ^61^, ^62^, ^63^, ^64^, ^65^, ^66^, ^67^, ^68^, ^69^, ^70^, ^71^, ^72^, ^73^, ^74^, ^75^, ^76^, ^77^, ^78^, ^79^, ^80^, ^81^ ^]^ Inset images: Thermoelectric parameter coupling and grain boundary scattering: reproduced with permission.^[^ ^40^ ^]^ Copyright 2021, Elsevier; and ion/electron thermal potential: reproduced with permission.^[^ ^41^ ^]^ Copyright 2021, American Chemical Society.

Appropriate TE materials should fulfill the following requirements: 1) high *zT* near room temperature, 2) excellent flexibility, and 3) sufficient mechanical strength or strong interaction with textile materials. This section summarizes and discusses the design of TETs from the aspect of TE materials selection. **Figure** **3** summarizes the reported maximum power factor (PF) and *zT* of TE materials, from which some state‐of‐the‐art organic‐based TE materials with excellent flexibility have exhibited competitive performance compared to inorganic TE materials.

### 3.1 Organic TE Materials

In recent years, conducting polymers have been frequently used in TETs than other organic TE materials. Hence, we mainly review recent advances in conducting polymers that are applicable to TETs, such as polypyrrole (PPy),^[^ ^82^ ^]^ polyaniline (PANI),^[^ ^83^ ^]^ poly (3,4‐ethylenedioxythiophene):poly (styrene sulfonate) (PEDOT:PSS),^[^ ^84^ ^]^ and so on. Among these conducting polymers, PEDOT:PSS with good hydrophilicity, chemical stability, and processability has been considered as one of the most popular p‐type candidates,^[^ ^8^, ^85^ ^]^ showing excellence for TETs because of their strong interactions with natural textile materials such as cotton, silk, wool, etc. The *zT* value of PEDOT:PSS has been reported to be ≈0.2–0.4, which stands out among other organic materials but is still lower than that of organic ones.^[^ ^86^ ^]^ The low *zT* of conducting polymers and the lack of n‐type organic TE materials still hinder their applications in designing high‐performance and intrinsically flexible TETs.^[^ ^11^, ^53^ ^]^

For pure conducting polymer‐based TE materials, the TE performance is mainly dependent on the PF = *S* ^2^ *σ*, due to their relatively low *k* (0.1–1 W m^−1^ K^−1^).^[^ ^87^ ^]^ At present, there are two main strategies to enhance the TE performance of conducting polymers including tuning doping and oxidation level via thermoelectric parameter coupling (Figure 3a)^[^ ^48^ ^]^ and nanostructuring.^[^ ^88^ ^]^ Bubnova et al. for the first time reported the *zT* of PEDOT:tosylate (Tos) reaching to ≈0.25 at room temperature, by regulating the post‐treatment time of doping agent, tetrakis(dimethylamino)ethylene (TDAE), to accurately control the doping level.^[^ ^50^ ^]^ Moreover, organic polar solvents or other acids have been widely used as secondary dopants to improve TE performance of conducting polymer‐based TE materials, such as dimethyl sulfoxide (DMSO), ethylene glycol (EG), diethylene glycol, *N*,*N*‐dimethylformamide, and sulfuric acid (H_2_SO_4_).^[^ ^87^, ^89^ ^]^ They can efficiently remove excess insulating components to optimize conducting polymer chain alignment toward high carrier mobility and density of sates (DOS) near Fermi level, thus *σ* and *S*, respectively. For example, Fan et al. fabricated PEDOT:PSS film with a *zT* of 0.49 through sequential post‐treatments with H_2_SO_4_ and sodium hydroxide (NaOH). It is believed to be attributed to high carrier mobility caused by acid treatment and optimized carrier concentration due to base treatment.^[^ ^49^, ^90^ ^]^ On the other hand, nanostructuring is another potential strategy to obtain high‐performance organic TE materials. Similarly, it can improve *S* by increasing DOS near the Fermi level (*E*_F_) and enhance carrier mobility and thus *σ* via improving crystallinity and alignment of conducting polymer chains.^[^ ^90^, ^91^, ^92^ ^]^ Cho et al. synthesized single‐crystal PEDOT nanowires (NWs) with ultrahigh carrier mobility of 88.08 cm^2^ V^−1^ s^−1^ and *σ* of ≈8797 S cm^−1^ by liquid‐bridge‐mediated nanotransfer printing with vapor phase polymerization (VPP).^[^ ^93^ ^]^

In addition, conducting polymer treated with ionic liquid (IL) has recently attracted attentions due to its high *S* due to the synergetic influence of Soret effect and doping and reconfiguration of conducting polymers (Figure 3b).^[^ ^94^ ^]^ For instance, the ion accumulation on surface significantly increases *S* of PEDOT:PSS by 1.2‐ to twofold, contributing to high *zT* of ≈0.75 at room temperature.^[^ ^46^ ^]^ However, ionic–electronic conducting polymers may not be directly used in TETs because they directly contact with human body may cause safety issue. Appropriate encapsulation should be considered to ensure wearing safety.

### 3.2 Inorganic TE Materials

**Figure 4:**
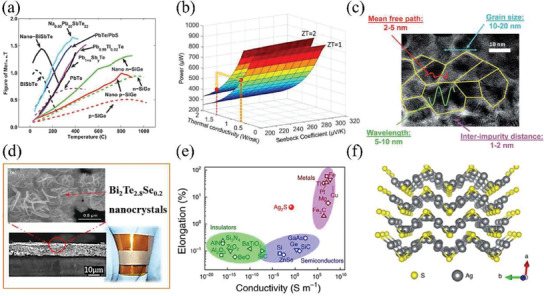
a) The summary of *zT* values in bulky and nanostructured inorganic semiconductors. Reproduced with permission.^[^ ^23^ ^]^ Copyright 2011, Royal Society of Chemistry. b) 3D illustration of power generation on human body as functions of *S* and *k* for TE devices with the same thickness in conditions of *zT* = 1 and *zT* = 2, respectively. Reproduced with permission.^[^ ^97^ ^]^ Copyright 2020, Elsevier. c) TEM image of a heavily doped Si_80_Ge_20_ nanocomposite along with some significant calculated characteristic lengths. Reproduced with permission.^[^ ^99^ ^]^ Copyright 2009, Royal Society of Chemistry. d) SEM images and photos of flexible Bi_2_Te_2.8_Se_0.2_ films on PI film. Reproduced with permission.^[^ ^100^ ^]^ Copyright 2016, Springer Nature. e) The elongation changes with respect to *σ* for *α*‐Ag_2_S and others.^[^ ^32^ ^]^ f) Crystal structure of *α*‐Ag_2_S along the [001] plane. Reproduced with permission.^[^ ^32^ ^]^ Copyright 2018, Springer Nature.

Inorganic TE materials usually have high *zT* >1 at high temperature (**Figure** **4**a).^[^ ^95^ ^]^ Among them, telluride (Te)‐based materials, especially Bi_2_Te_3_‐based alloys, show great potentials for wearable TE devices, owing to their high *zT* and structural stability near room temperature.^[^ ^1^, ^60^ ^]^ At present, p‐type and n‐type Bi_2_Te_3_ can achieve outstanding *zT* values of 1.3–1.8 and 1.0–1.4 near room temperature, respectively.^[^ ^96^ ^]^ Unlike organic materials, inorganic materials with ordered cell structure always have high *k*, which may reduce the self‐built temperature difference across TE devices, resulting in limited power output. Meanwhile, Nozariasbmarz et al. found that the TE material with lower *k* but comparable *zT* can generate higher power on human body than that with high *S* (Figure 4c).^[^ ^97^ ^]^ So how to suppress *k* without sacrificing PF is essential to enhance the output performance of inorganic‐based TE devices. On the other hand, inorganic atoms are bonded by strong covalent or ionic–covalent bonds with directivity and saturability, which leads to high elastic modulus and unfavorable slipping process.^[^ ^98^ ^]^ Hence, inorganic materials are typically fragile and rigid at room temperature. Thus, in this section, we will review the improvement in TE performance and flexibility of inorganic materials for their potential in the field of wearable devices, especially TETs.

Manipulating doping level or involving different dopants are common methods to improve TE performance in inorganic semiconductors.^[^ ^101^ ^]^ Se, Sn, Ag, Cu are usually used as p‐type dopants,^[^ ^102^ ^]^ while AgI, CuI, SbI_3_, and CuBr_2_ can act as n‐type ones.^[^ ^103^ ^]^ BiTeSe‐ and BiSbTe‐based alloys are the most successful p‐type and n‐type Te‐based semiconductors, respectively. They can achieve high *zT* of 1.01–2.4 near room temperature by controlling the stoichiometric ration of Bi, Te, and Sb elements and optimizing their doping level.^[^ ^104^ ^]^ In inorganic semiconductors, the contribution of lattice thermal conductivity (*k*_l_) in total *k* usually surpasses the electronic contribution.^[^ ^105^, ^106^ ^]^ *k*_l_ lies on the crystal structure and lattice parameters, which can be significantly reduced by incorporating nanophases with mismatched lattice with enhanced phonon scattering. For example, Dou et al. incorporated amorphous SiO_2_ nanoparticles into (Bi_2_Te_3_)_0.2_(Sb_2_Te_3_)_0.8_ bulk, leading to the reduction of *k* to ≈0.46 W m^−1^ K^−1^ and high *zT* of 1.12 at 303 K, owing to enhanced phonon scattering at nanoparticles and phase boundaries.^[^ ^62^ ^]^ Nanostructuring is another way to enhance phonon scattering for high *zT* via introducing grain boundaries and defects in nanostructured inorganic bulk materials (Figure 4c) or reducing dimensions of bulk into microfibers, nanofiber, or even quantum fibers.^[^ ^107^, ^108^ ^]^ For example, Tang et al. fabricated Bi_2_Te_3_ bulk (*zT* ∼ 1.35 at 300 K) with layered nanostructure via melt spinning and spark plasma sintering.^[^ ^61^ ^]^ Meanwhile, it has been proposed that nanostructuring can significantly increase the flexibility of inorganic materials, because of the reduced bending stiffness. Varghese et al. reported flexible Bi_2_Te_2.8_Se_0.2_ nanocrystal‐based films with *zT* of 0.43 at room temperature using screen printing (Figure 4d). After 150 bending cycles, the electrical resistance of film showed insignificant change of only 4.5% at bending radius of 5 mm.^[^ ^100^ ^]^

In recent years, more and more attentions have been paid to emerging ductile inorganic semiconductors, such as Ag_2_S and Ag_2_Se as reported by Chen's research group, due to their promising plasticity and flexibility.^[^ ^109^ ^]^ The extraordinary ductility can protect inorganic semiconductors from destructive fractures under external impact, showing great potentials to design flexible and deformable TETs. For example, *α*‐Ag_2_S with capability of plasticity deformation shows engineering strains of ≈4.5% in tension, 50% in compression, and >20% in three‐point bending, which is comparable to that of metals (Figure 4e).^[^ ^32^ ^]^ These properties are believed to be attributed to its structural and chemical bonding (Figure 4f). The weak bonding between Ag_2_S layers contributes to small slipping energy and large cleavage energy, leading to the slipping without cleavage. In the slipping process, some bonds vanish while new bonds form subsequently, which are as strong as the original Ag—S bonds between and within layers.^[^ ^98^ ^]^ Based on the first‐principle calculation and Boltzmann transport theorem, *zT* values of p‐type and n‐type Ag_2_S in the *Z*‐direction are expected to be 0.97 and 1.12 at room temperature.^[^ ^110^ ^]^ For Ag_2_Se, it has been deemed to be promising as n‐type candidate for wearable TE devices since its *zT* can be up to 1.2 at room temperature, which outperforms brittle Bi_2_Te_3_.^[^ ^111^ ^]^ Jiang et al. developed flexible Ag_2_Se NWs film with ultrahigh PF ∼ 1882 µW m^−1^ K^−2^ at 300 K via vacuum‐assisted filtration and hot pressing.^[^ ^112^ ^]^ After 1000 bending cycles, the PF for Ag_2_Se NWs films were maintained to be 90.7% with a bending radius of 4 mm and possessed tensile strain of ≈5%. More importantly, S, Se, and Ag elements used in ductile inorganic semiconductors are abundant in resource and low in toxicity, which is crucial to fabricate environmental benign flexible TE devices at low cost and large scale.^[^ ^113^ ^]^

### 3.3 Carbon Nanocrystal‐Based TE Materials

Over the same period with conducting polymers, carbon nanocrystals also have received considerable interests as light‐weight and flexible TE materials for wearable applications, owing to their high *σ*, tunable TE properties, excellent flexibility, and mechanical properties. Moreover, they can be easily integrated with other TE materials as second phase or directly assembled into carbon‐based fibers via spinning for TET fabrication. The major scientific issue may be their high anisotropic thermal conductivity among all types of TE materials.

Among them, carbon nanotubes (CNTs) with PF of ≈700–1000 µW m^−1^ K^−2[^ ^114^ ^]^ possessing quasi‐1D structure, have been applied to fabricated fiber‐based TETs. Pristine CNT is intrinsically n‐type semiconductor but often doped to p‐type under ambient atmosphere because of oxygen doping. But they can be easily doped into n‐type by simple chemical doping.^[^ ^57^ ^]^ For instance, Chen et al. synthesized n‐type single‐walled CNTs (SWCNTs) with high air‐stability via doping with alkylammonium cationic surfactant (CTAB).^[^ ^58^ ^]^ *S* and *σ* of CTAB‐doped SWCNTs were −47 µV K^−1^ and 840 S cm^−1^, respectively. Regarding the processability, pristine CNT has poor dispersibility in solvents because of its high surface energy^[^ ^115^ ^]^ and hydrophobic surface, which can be addressed by treatment using surfactants for improved dispersion in mixing with other TE materials.^[^ ^116^ ^]^

Graphene is another type of frequently used carbon nanocrystal, which is a monolayer of carbon atoms with regular hexagonal honeycomb lattice.^[^ ^11^ ^]^ It is the thinnest ever known low‐dimensional material and the strongest ever measured.^[^ ^117^ ^]^ Graphene possesses high carrier mobility due to the weak electron–phonon interaction. Nevertheless, their *zT* are still very low because of their high *k* but small *S* arising from their gapless band structure in metallic feature.^[^ ^118^, ^119^, ^120^ ^]^ Some reports proposed that their *zT* can be enhanced by converting it into 1D graphene nanoribbons with lower *k* than that of graphene.^[^ ^121^, ^122^ ^]^ Owing to its unique mechanical, thermal, and optical properties, graphene has been widely studied for textile‐based electronics applications.^[^ ^123^, ^124^ ^]^ Similar to CNT, graphene is also hydrophobic, showing poor dispersibility in water and other solvents, which limits the combination of graphene with textile.^[^ ^34^ ^]^ Besides surface treatments with ionic surfactants and ILs, surface oxidation has also been frequently used to functionalize graphene to improve dispersion stability.^[^ ^124^, ^125^, ^126^ ^]^ Overall, the thermoelectric properties of carbon nanocrystals are poor but they can work as effective secondary phase in making hybrid TE materials.

### 3.4 Hybrid TE Materials

#### 3.4.1 Organic/Inorganic TE Composites

Rationally designed organic/inorganic composites can deliver exciting electrical and mechanical properties compared to each component, owing to the combination of flexibility of organics and high TE properties of inorganics. Physical mixing, in situ polymerization, layer‐by‐layer self‐assembling are usually used to fabricate inorganic/organic TE composites.^[^ ^11^, ^127^ ^]^ However, it is worth noting that the direct mixing of organics and inorganics is not necessary to offer high performance and mechanical flexibility. Rationally manipulating the interfaces between organics and inorganics should be employed to leverage the intrinsic flexibility of organic component and maintain the desired TE properties of inorganic fillers.^[^ ^128^ ^]^ For instance, low‐dimensional fillers with large interfacial energy can accelerate the alignment of polymer chains along fillers and thus optimize the configuration of conducting polymer chains, facilitating carrier transport at interfaces.^[^ ^23^, ^91^ ^]^ For example, Coates et al. first observed *σ* and PF of PEDOT:PSS/Te NWs composites surpassed individual component because PEDOT:PSS coating on Te NWs displayed ordered arrangement of polymer chains compared to bulky PEDOT:PSS.^[^ ^54^ ^]^ Furthermore, the energetic mismatches at the interface between polymer matrix and inorganic fillers can offer selective carrier scattering, leading to high *S* and PF. For example, PEDOT/Bi_2_Te_3_ nanoparticles hybrid with monodispersed and periodic nanophase, which may introduce interfacial barriers, yield *zT* of 0.58, which is much higher than most of the organic or hybrid TE materials.^[^ ^51^ ^]^ Shi et al. reported a PEDOT‐Te quantum dot composite film with enhanced PF up to 100 µW m^−1^ K^−2^ when introducing only 2.1–5.8 wt% Te quantum dot (<5 nm) and they found the effect of Te particles size on the increase of *S* is prominent, which can be explained via a phonon drag theory.^[^ ^129^ ^]^ The small‐sized particles with diameter below 2 nm can effectively reduce phonon scattering below the phonon frequency at room temperature (*T* ≈ 293 K).

On the other hand, the incorporation of inorganic semiconductors also can fabricate n‐type TE hybrid materials. For example, We et al. demonstrated n‐type PEDOT:PSS/Bi_2_Te_3_ nanosheets hybrids using screen printing with *zT* of 0.16 at room temperature.^[^ ^56^ ^]^ Wang et al. obtained flexible n‐type CNT/PEDOT hybrid film with *zT* of 0.5 via in situ polymerization and TDAE post‐treatment.^[^ ^130^ ^]^ Recently, Li et al. reported a flexible Ag_2_Se/Se/PPy composite film with ultrahigh PF of 2240 µW m^−1^ K^−2^ at 300 K due to energy filtering effect at the hetero‐interface of Ag_2_Se/Se and Ag_2_Se/PPy in the film.^[^ ^77^ ^]^

#### 3.4.2 Polymer Blends

Unlike organic/inorganic TE composites, conducting polymer blends are promising intrinsically soft and flexible TE materials. Polymer blends are typically composed of one polymer with low *S* and high *σ* and another polymer with high *S* and low *σ* and the peak value of PF can be achieved via the trade‐off between *S* and *σ*. Another possible way to improve PF of conducting polymer blends is to blend polymers with different energetic distribution of states. Chen et al. observed that the PF of PPy NWs/PEDOT NWs blends was increased significantly by carefully turning the energetic structure of PPy NWs (nanofillers), due to a built‐in energy barrier at nanowires interface.^[^ ^131^ ^]^

**Figure 5:**
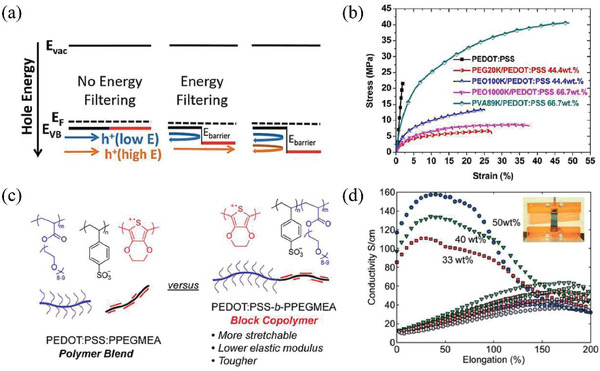
a) Illustration of band alignment at interfaces between organics and inorganics for possible energy filtering effect. Reproduced with permission.^[^ ^138^ ^]^ Copyright 2017, Royal Society of Chemistry. b) Typical stress–strain curves of neat PEDOT:PSS and various PEDOT:PSS‐based polymer blends. Reproduced with permission.^[^ ^139^ ^]^ Copyright 2015, American Chemical Society. c) Schematic of polymer blends and block copolymers. Reproduced with permission.^[^ ^137^ ^]^ Copyright 2018, American Chemical Society. d) The electrical conductivity versus elongation of PEDOT/PU composites with different contents of PEDOT. Reproduced with permission.^[^ ^136^ ^]^ Copyright 2007, Wiley.

Intrinsic conducting polymer is a kind of flexible but relatively fragile TE materials due to the limited *π*‐electron delocalization in conjugated chains caused by the electrostatic interactions between charged polymers and molecular/counterions.^[^ ^27^, ^119^, ^120^, ^121^ ^]^ In contrary to aforementioned ones, polymer blends with intrinsic deformability and potential high TE performance have not earned attentions, which may contribute to enhance the deformability and comfort of TETs.^[^ ^131^, ^132^, ^133^ ^]^ The general strategy to develop intrinsically stretchable conducting polymers‐based TE materials is to incorporate conducting polymers with elastomers. Generally, conducting polymers can be dispersed in solvents at room temperature that allows them be facilely processed by various all‐solution‐processable methods.^[^ ^134^ ^]^ Hence, it would be better if polymer elastomers are soluble in water or polar solvents. For instance, polyurethane (PU), poly(ethylene glycol), poly(ethylene oxide) (PEO), poly(ethylene glycol)‐block‐poly(propylene glycol)‐block‐poly(ethylene glycol) triblock copolymer (PEO‐PPO‐PEO), and poly(vinyl alcohol) (PVA) with excellent solubility in solvents are good choices (**Figure** **5**b).^[^ ^135^ ^]^ Hansen et al. reported PEDOT/PU blends with *σ* up to 160 S cm^−1^ under 50% strain loading (Figure 5d).^[^ ^136^ ^]^ Additionally, the introduction of soft segments into conducting polymers has been proved to offer the stretchability of TE materials at microstructure level. Kayser et al. obtained an intrinsically stretchable PEDOT:PSS film^[^ ^137^ ^]^ with *σ* of 14.8 S cm^−1^ by introducing soft segments of poly(poly(ethylene glycol) methyl ether acrylate) (PPEGMEA) (Figure 5c).^[^ ^137^ ^]^ The PEDOT:PSS‐*b*‐PPEGMEA hybrids withstood elongation of 128% and toughness of 10.1 MJ m^−3^.

### 3.5 Interaction between TE Materials and Textile Fibers

**Figure 6:**
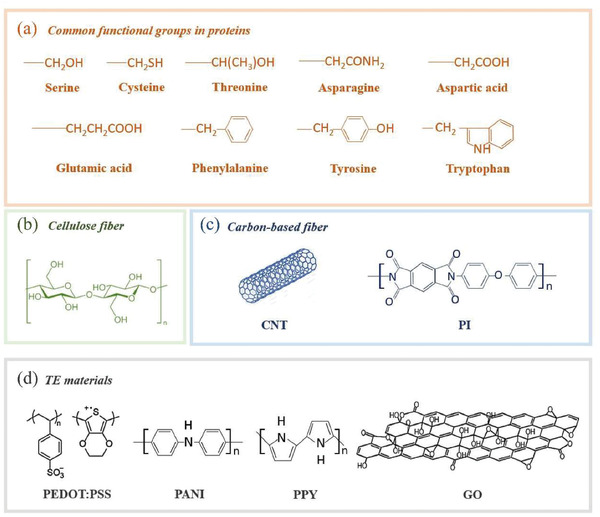
Chemical structures of a) common functional groups in protein fiber, b) cellulose fiber, c) carbon‐based fiber, and d) some conducting polymers and GO.

In this section, we will emphasize the interaction between TE materials and textile fibers, which is crucial to design TET with the best match of TE materials and textile fibers. The strong interaction between TE materials and textile fibers is essential to improve their abrasive resistance, allowing further textile processing and daily use. The interaction between TE materials and textile fibers can significantly affect the TE performance and mechanical stability of their hybrids. In general, protein fibers, plant fibers, chemical fibers including high‐performance fibers are usually used for making TE fibers or TETs (**Figure** **6**). The interfacial interaction mainly depends on the surficial microstructure and chemistry of textile fibers.^[^ ^140^, ^141^ ^]^

The physical interaction between fibers and TE materials can be enhanced by introducing microstructure to enlarge the specific surface area of fibers. Xu et al. found that the regenerated cellulose fibers, Coolmax, with trench and groove surface can absorb more graphene oxide (GO) thus high electrical conductivity than cotton and wool fibers due to the large specific surface area of Coolmax fiber that can enhance its interaction with GO.^[^ ^142^ ^]^

**Figure 7:**
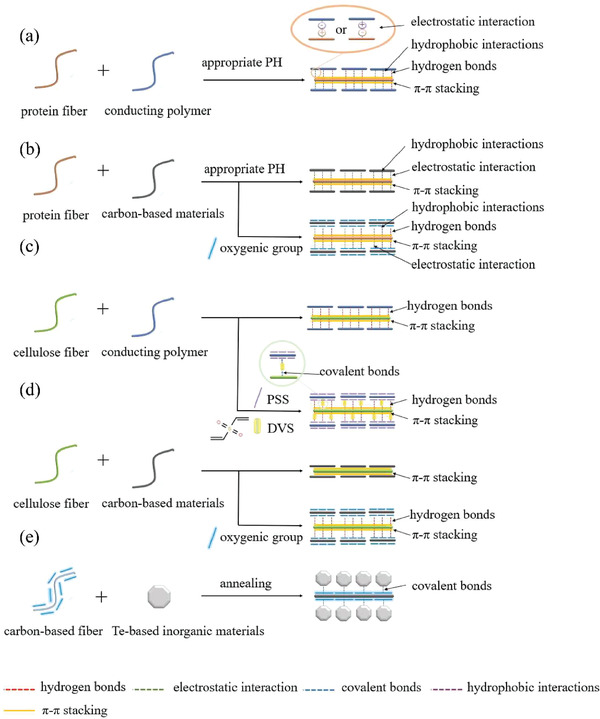
Mechanism underlying the formation of interaction between a) cellulose fiber and conducting polymer, b) cellulose fiber and carbon‐based materials, c) protein fiber and conducting polymer, d) protein fiber and carbon‐based materials as well as e) carbon‐based fiber with oxygenic groups and tellurium‐based inorganic semiconductors.

Natural animal fibers mainly made of protein have been widely studied in wearable electronic devices owing to their outstanding biocompatibility.^[^ ^143^ ^]^ Based on the nature of proteins, the protein fiber will carry positive charge when the pH value is higher than its isoelectric point, otherwise they will be negatively charged. Hence, the strong adhesion between TE materials and protein fibers can be realized by the formation of electrostatic interactions (**Figure** **7**a,b)^[^ ^144^, ^145^ ^]^ during dyeing process with proper pH value. For example, Ryan et al. discovered that PEDOT:PSS can be successfully bonded to silk fibers at acidic environment (pH ∼ 2). The electrostatic interaction is built between negatively charged butane sulfonate side chains of PEDOT:PSS and positively charged silk fibers.^[^ ^146^ ^]^ Interestingly, it has been observed that the silk fiber can be slightly dyed by PEDOT:PSS at alkaline condition with pH ∼ 11.^[^ ^147^ ^]^ According to the chemical structure of proteins (Figure 6a), *π*–*π* interaction and hydrophobic interactions have been proposed to the contribution of the binding of PEDOT:PSS to various protein‐based structures under alkaline conditions.^[^ ^148^, ^149^ ^]^ Meanwhile, some researchers found that the conjugation of polymers can also affect their potential to adhere protein fibers.^[^ ^147^, ^150^ ^]^ For PANI or PPy, there are numerous primary and secondary amino groups in their molecules (Figure 6d). Therefore, hydrogen bonding interaction between the oxygen atoms of peptide linkages and hydrogen atom on amino group in PANI (or PPy) is suggested to be the dominant interaction while the electrostatic interaction may play a secondary role.^[^ ^151^ ^]^ For PEDOT, the electrostatic interaction is crucial, owing to its sulfur atom with positive charge on the five‐membered heterocyclic monomer.^[^ ^152^ ^]^ In general, GO presents electronegativity in aqueous solutions because of its abundant oxygen‐rich functional groups and thus it can be easily adsorbed with the amino group of silk by the electrostatic interaction at low pH <4 (Figure 7b).^[^ ^153^ ^]^

Natural plant fibers, especially cotton fibers, are commonly used to fabricate conducting fabrics because of their comfort, resource abundance, durability, and regeneratable feature.^[^ ^154^ ^]^ The major constituent of plant fibers is cellulose with abundant oxygenic groups and benzene ring (Figure 6b), which can be beneficial to their interaction with TE materials by forming hydrogen bonds and *π–π* stacking (Figure 7c,d).^[^ ^155^, ^156^ ^]^ Alamer et al. observed the formation of hydrogen bonding between PEDOT:PSS and cotton fibers via thermal gravimetric analysis. They also found that hydrogen bonding can improve the stereoregularity of cellulose matrix and further increase the length of polymer chains. Cai et al. developed a rGO‐coated cotton fabric via a “dip and dry” method and heat treatment,^[^ ^157^ ^]^ leading to good mechanical stability due to strong interaction between rGO and cotton fibers. Moreover, crosslinking agents were also added into polymer solution to further improve the adhesion of conducting polymer to the hydroxy‐rich cellulose fibers. Recently, a novel crosslinker, divinyl sulfone (DVS) with two active sits, attracts a lot of attentions. For example, it can improve the binding force between PEDOT:PSS and cotton fibers.^[^ ^158^, ^159^ ^]^ Two vinyl reactive ends in DVS (Figure 6f) can replace the hydrogen in hydroxyl, present on cellulose fiber and PSS via Oxa‐Michael nucleophilic addition followed with the formation of covalent crosslink within PSS and covalent bonds with cellulose surfaces (Figure 7c).^[^ ^160^ ^]^ Meanwhile, DVS, like DMSO or EG, can act as second dopant to improve *σ*.^[^ ^161^ ^]^ Though there is lack of understanding on the applicability of DVS on other TE materials, however, according to the principle of Oxa‐Michael nucleophilic addition, we expect that DVS should be applicable to TE materials with certain electronegativity, such as GO, PPy:PSS, and so on.

The chemical components and microstructure of chemical fibers can be easily controlled. The adhesion between organic materials and chemical fibers can be strengthen by introducing functional groups containing nitrogen or oxygen (i.e., carboxyl, hydroxy, amido, etc.). Tzounis et al. reported that CNTs can be uniformly coated on the surface of amine‐functionalized glass fiber yarns with covalent bonds.^[^ ^162^ ^]^ Meanwhile, adhesive agents can also be used to facilitate the effective combination of TE materials and chemical fibers. For example, bovine serum albumin (BSA), an electrostatic glue, is an amphiphilic protein with featured hydrophobic and hydrophilic patches, allowing it to adhere to organic materials and inorganic materials via nonpolar, polar, and ionic interactions.^[^ ^163^, ^164^ ^]^ Yun et al. reported BSA can improve the absorption of GO onto the surface of nylon fibers toward highly stable rGO/BSA/nylon textiles under cyclic bending, temperature, and washing treatments.^[^ ^165^ ^]^

For inorganic TE materials, high‐performance fibers with high‐temperature resistance are preferred, owing to their thermal stability, however, the inorganic semiconductor coating layers on them are peculiarly prone to delamination and fragmentation under bending and abrasion conditions due to weak interaction, referring to Van der Waals force, and mismatched mechanical properties. Interestingly, some new bonds have been observed at the interface between inorganic materials and fibers after high‐temperature sintering, which provides opportunities to enhance the durability of inorganic‐based TETs (Figure 7e). Kim et al. reported a possible interaction between oxygen functional groups on CNTs and Te atoms of Bi_2_Te_3_ after sintering, and speculated oxygen atoms at the interface between CNTs and Bi_2_Te_3_ can be regarded as binding agent.^[^ ^166^ ^]^ Recently, Zheng et al. reported the formation of covalent bond between Bi_2_Te_3.3_Se_0.2_ and polyimide (PI) filament after sintering, which may contribute to mechanical stability under bending deformation.^[^ ^167^ ^]^

## 4 Scalable Fabrication of TE Fibers/Yarns

The TE fibers/yarns are the basic unit to fabricate TETs with textile manufacturing processes including embroidery, weaving, or knitting. They can be categorized into p‐type TE fibers/yarns, n‐type TE fibers/yarns, and p–n segmented TE fibers/yarns. The TE and mechanical performances of TE fibers/yarns is the synergetic effect of materials engineering and textile process. In this section, we will introduce the major state‐of‐the‐art fabrication techniques for the fabrication of TE fibers/yarns, analyze their processing requirements, and compare their advantages and disadvantages.

### 4.1 Single p‐Type or n‐Type TE Fibers/Yarns

**Figure 8:**
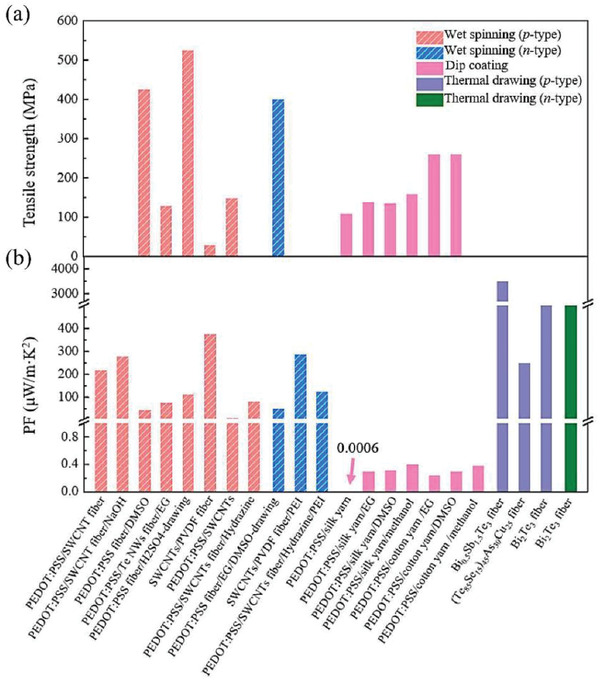
A summary of TE and mechanical properties of TE yarns: a) tensile strength and b) PF.

The p‐type or n‐type TE fibers/yarns can be fabricated by various methods including wet spinning, gelation spinning, thermal drawing, solution casting, thermal evaporation, and magnetron sputtering. But some of them may not be able to fabricate TE fibers/yarns at scale, such as thermal evaporation and magnetron sputtering, which will not be discussed in this section. **Figure** **8** summarizes the PF and tensile strength of advanced TE fibers/yarns. We can see that TE filaments fabricated by wet spinning usually show excellent TE properties and mechanicalperformance. And to provide convenience to readers to select an appropriate technolody, advantages and disadvantages of varioufabrication methods of TE fibers/yarns are also summarized in **Table** **1**.

**Table 1:** The advantages and disadvantages of different fabrication methods of TE fibers/yarns

Methods	Advantages	Disadvantages
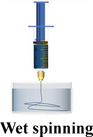 Wet spinning	♦ Simple process ♦ Relatively high TE properties ♦ Large‐scale production ♦ p–n segmented TE fibers	♦ Poor controllability
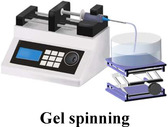 Gel spinning	♦ Simple process ♦ Ultrahigh tensile strength ♦ Good durability ♦ p–n segmented TE fibers	♦ Low TE properties ♦ Strict processing conditions
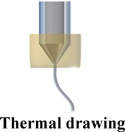 Thermal drawing	♦ Ultrahigh TE properties ♦ Good durability	♦ Strict processing conditions ♦ Relatively low flexibility
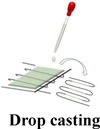 Drop casting	♦ Simple process ♦ Good controllability ♦ Low cost ♦ p–n segmented TE fibers	♦ Relatively low TE properties ♦ Poor durability
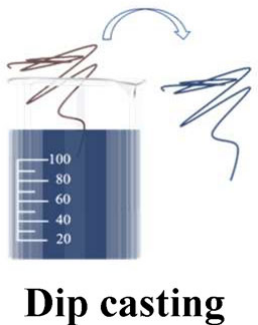 Dip casting	♦ Simple process ♦ Large‐scale production ♦ Low cost	♦ Relatively low TE properties ♦ Poor durability

*Wet spinning* is a widely used method to manufacture continuous polymer‐based filaments in traditional textile industry. It also has been adopted to spin conducting filaments with conducting dopes, such as GO,^[^ ^168^, ^169^ ^]^ CNT,^[^ ^170^, ^171^ ^]^ PEDOT:PSS,^[^ ^172^, ^173^, ^174^ ^]^ PANI,^[^ ^175^ ^]^ etc., which is applicable to fabricate TE filaments. Typically, the TE dope is first dissolved or dispersed in solvent to form a uniform solution or dispersion, and then extruded into a coagulation bath through a needle spinneret to solidify into filaments. The spinning scalability and TE/mechanical properties of TE filaments largely depend on spinning conditions, including the kind of coagulation bath,^[^ ^172^ ^]^ rheological characteristics of spinning dope,^[^ ^176^ ^]^ the needle geometry, and post‐treatment.^[^ ^177^ ^]^ **Table** **2** summarizes the influence of coagulation bath and post‐treatment on TE and mechanical properties of TE filaments. Wen et al. found that reducing the inner diameter of needle in a limited range (<30 G) can improve *σ* of fibers with unchanged *S*, due to the improved polymer chain alignment raised by spatial confinement.^[^ ^178^ ^]^ Ionic surfactants can be used to improve the dispersibility of CNTs and tune the doping level and type of CNT filaments. Mukai et al. successfully made a raw CNTs fiber with the aid of sodium cholate (SC), in which *σ* and tensile strength were optimized to ≈14 284 S cm^−1^ and ≈887 MPa, respectively, which is believed to be ascribed to the enhanced CNT orientation along filament.^[^ ^179^ ^]^ On the other hand, post‐treatments with organic solvents, acid/base, or salt solution contribute to obtain high‐performance TE filaments by modifying the doping level, orientation, and crystallinity of polymers. Sarabia‐Riquelme et al. developed highly conductive wet‐spun PEDOT:PSS filaments through inclusion of a H_2_SO_4_ drawing step and immersing obtained filaments in H_2_SO_4_ for additional time. The filament achieved PF and break stress of ≈115 µW m^−1^ K^−2^ and 550 MPa, respectively, by owing to the ultrahigh crystallinity of PEDOT:PSS via *π*–*π* stacking.^[^ ^173^ ^]^ Nevertheless, strong and corrosive acid/base solvents may give rise to safety and environmental issues and are undesirable in commercial fabrication.^[^ ^180^, ^181^ ^]^ Zhou et al. reported wet‐spun PEDOT:PSS fibers with *σ* of 2804 S cm^−1^ and tensile strength of 409.8 MPa by EG treatment and hot‐drawing. The synergetic enhancement was proved to be owing to the alignment of PEDOT chains.^[^ ^182^ ^]^ Additionally, some studies have involved inorganic materials into conducting polymers to spin hybrid TE filaments. For example, flexible PEDOT:PSS/Te NWs fiber exhibited PF of 78 µW m^−1^ K^−2^ and tensile strength of 130 MPa.^[^ ^183^ ^]^ Apart from TE properties, wearable devices also provide requirements for the mechanical performance of TE filaments. It has been proposed that CNTs and cellulose nanofibrils (CNFs) can significantly improve the strength of conducting polymer‐based TE filaments without sacrificing their *σ*, due to high strength of fillers and strong *π–π* stacking between polymer and filler.^[^ ^177^ ^]^ Fall et al. reported PEDOT:PSS/CNFs filament with EG treatment, demonstrating tensile stiffness of 20 GPa, tensile strength 356 MPa, and *σ* of 150 S cm^−1^ simultaneously.^[^ ^184^ ^]^

**Table 2:** The wet‐spun TE filaments and their TE performance and mechanical properties

Sample	Coagulation bath	Post treatment	*S* [µV K^−1^]	*σ* [S cm^−1^]	PF [µW m^−1^ K^−2^]	Tensile strength [MPa]	Young's moduli [GPa]	Elongation [%]	Ref.
PEDOT:PSS/SWCNT filament	H_2_SO_4_	–	27.1	2982	219	–	–	–	[161]
PEDOT:PSS/SWCNT filament	H_2_SO_4_	NaOH	36.4	2110	280	–	–	–	[161]
PEDOT:PSS filament	IPA+10% DMSO	DMSO	15.5	2000	40–50	425	15.5	5	[156]
PEDOT:PSS filament	acetone/IPA	EG/hot‐drawing	–	2804	–	409.8	8.3	21	[166]
PEDOT:PSS/Te NWs filament	IPA	EG	56	248	78	130	6.5	–	[167]
PEDOT:PSS filament	IPA+10% DMSO	DMSO‐drawing	≈15	2244	50.49	400	16	5	[157]
PEDOT:PSS filament	IPA+10% DMSO	H_2_SO4‐drawing	18–19	3663	115	500–550	20–22	7.5	[157]
SWCNTs/PVDF filament	DI water	–	40.2	1950	378	30.1	–	10.4	[160]
SWCNTs/PVDF filament	DI water	PEI	−33.1	2050	289	–	–	–	[160]
PEDOT:PSS/CNFs filament	HCl	EG	–	154	–	356	19	4.1	[168]
PEDOT:PSS/SWCNTs	methanol	–	16.6	361	10.1	149	–	6.2	[155]
PEDOT:PSS/SWCNTs filament	methanol	hydrazine	29.3	979	83.2	–	–	–	[155]
PEDOT:PSS/SWCNTs filament	methanol	hydrazine/PEI	−48.1	540	125	–	–	–	[155]

Gel spinning, known as semimelt spinning, is an efficient method to fabricate synthetic polymer‐based filaments with high mechanical properties. The hot spinning solution or plasticized gel is ejected through the spinneret into low‐temperature coagulation bath to make polymer filaments in the gel state and then the new formed filaments are stretched by ultra‐high extension.^[^ ^185^ ^]^ Compared with the wet spinning, gel spinning requires polymers with high molecular weight because these polymers can support high‐magnification stretching. Hence, the gel spinning can fabricate high‐strength fibers with the following advantages:^[^ ^186^ ^]^ 1) increased molecular weight eliminates defects in filaments introduced by polymer chain groups. 2) High magnification stretching can reduce the entanglement of polymer chains and improve the crystallinity and orientation of polymer chains. For example, the polyacrylonitrile (PAN)/CNTs fibers by gel spinning achieved a rather high value of 5.8 and 375 GPa.^[^ ^187^ ^]^ Nevertheless, the poor TE performance remains the major issue for gel‐spun TE filaments due to the addition of a large amount of insulating polymers.^[^ ^188^ ^]^

Thermal drawing is a traditional method to fabricate optical fibers. It has become one novel strategy to fabricate inorganic TE filaments in recent years, which overcomes the insoluble issue of inorganic TE materials during other spinning processes. It has high requirements for vacuum level (10^−3^ Torr) and temperature (>1000 K). The inorganic TE filaments are fabricated from a macroscopic rectangular preform, which is composed of the inorganic TE materials core and protective cladding.^[^ ^189^ ^]^ Then the preform is consolidated at high temperature under vacuum and finally thermally drawn in a vertical tube furnace to obtain TE filaments in meter scale. Selecting appropriate cladding materials is crucial to the thermal drawing technology. The glass transition temperature of the cladding material should be slightly higher than the melting point of TE materials so as to withstand the draw stress and support the molten TE filler while continuously and controllably scaling down to microscopic dimension.^[^ ^190^ ^]^ The prepared TE filaments usually have high TE properties rivaling inorganic TE bulks and excellent durability whereas their flexibility is still restricted by intrinsic brittle nature of inorganic materials. For instance, the PF of p‐type Bi_0.5_Sb_1.5_Te_3_/borosilicate glass fibers and n‐type Bi_2_Se_3_/borosilicate glass fibers was 3.52 and 0.65 µW m^−1^ K^−2^, respectively.^[^ ^191^ ^]^ Nevertheless, the bending radius of inorganic fibers with a diameter of 50 µm was ≈1 cm.^[^ ^191^ ^]^

Coating is a sort of facile method to fabricate core–shell‐structured textile fiber‐based TE yarns/filaments, including drop casting and dip coating. Drop casting depends on the deposition of liquid drops with controlled sizes and momentum onto a substrate to obtain the coating of TE materials after the evaporation of solvent.^[^ ^192^ ^]^ Dip coating refers to immersing yarns/filaments into a solution containing TE materials followed by forcibly drying to form TE yarns/filaments.^[^ ^193^ ^]^ In some attempts, yarns/filaments were immersed into oxidant solution instead of polymer dispersion and then were exposed to organic monomer vapor to fabricate TE yarns/filaments by in situ VPP.^[^ ^194^ ^]^ Coating process has advantages of low cost, low energy consumption, and simple process. The majority of TE materials can be processed by this method including organic TE materials with good dispersibility and some inorganic TE nanomaterials.^[^ ^195^ ^]^ The coating layer of TE materials usually has negligible effect on the intrinsic mechanical performance of yarns/filaments. Thus, the TE yarns/filaments can maintain excellent flexibility and high mechanical strength.^[^ ^146^ ^]^ However, the coating quality determines the TE performance and durability of TE yarns/filaments. When the yarns/filaments show higher surface tension than that of coating droplets, the well‐binding force and high‐quality coating layer will be obtained. In Section 3.5, we discussed the interfacial interaction from the point of view of chemical structure of materials and found that the interfacial adhesion can be reinforced by introducing active functional groups on fibers or using adhesive agents with polar and nonpolar groups.^[^ ^163^, ^165^, ^196^ ^]^ Some researchers reported functionalized fiber‐based substrate by chemical treatment,^[^ ^162^ ^]^ plasma treatment,^[^ ^197^ ^]^ and UV‐light treatment^[^ ^198^ ^]^ in order to improve the surface tension of substrates. In addition, some other operations, such as increasing the soaking times/numbers,^[^ ^199^ ^]^ adjusting the pH value of dispersion,^[^ ^147^ ^]^ improving dyeing temperature,^[^ ^200^, ^201^ ^]^ and applying external pressure,^[^ ^202^ ^]^ also have been proved to enhance the loading volumes of TE materials on fibers, which is helpful to keep high TE properties and durability (Table 1). However, for given TE materials, the coating methods usually result in lower TE performance of TE yarns/filaments fabricated than that of TE filaments by other spinning or thermal drawing methods (Figure 8).

### 4.2 p–n Segmented TE Filaments

Aforesaid single p‐type or n‐type TE fibers require further manual electrical connection to fabricate TEGs. However, the electrical contact resistance between TE fibers and interelectrodes is considerably high that significantly limits the TE performance and scalability of TETs.^[^ ^203^ ^]^ The emerging p–n segmented TE filaments can well solve this issue. For p–n segmented TE filaments, the p‐type and n‐type fiber components arranged periodically are electrically connected in series along TE filaments. They can be directly used as weaving units to manufacture TETs or integrated into 3D fabrics to harvest heat energy in the out‐of‐plane direction while maintaining the aesthetic and comfortable features of garments. The scalable manufacture methods of p–n segmented TE filaments meet the demands of long‐term development and can widely broaden the real‐world applications of TETs.

**Figure 9:**
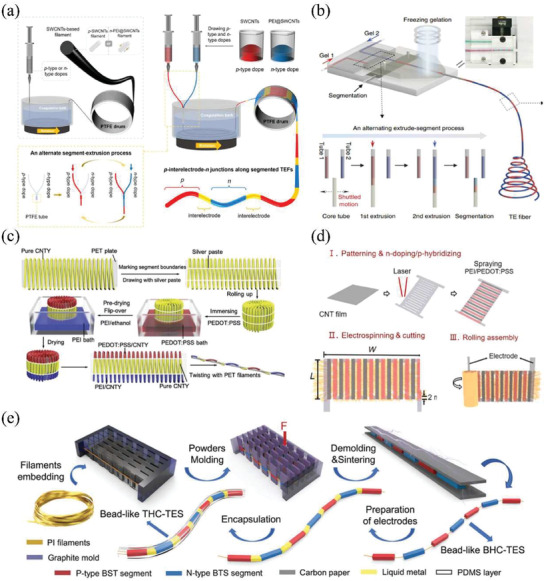
Illustration of the fabrication of p–n segmented TE fibers/yarns. a) The scalable alternate wet‐spinning process of p–n segmented TE filaments. Reproduced with permission.^[^ ^204^ ^]^ Copyright 2022, American Chemical Society. b) The continuously alternating extrusion process of gel spinning. Reproduced with permission.^[^ ^188^ ^]^ Copyright 2020, Springer Nature. c) The scalable dip coating process of p–n segmented TE yarns.^[^ ^191^ ^]^ d) The process for fabrication and rolling assembly of patterned CNT film. Reproduced with permission.^[^ ^205^ ^]^ Copyright 2022, Elsevier. e) The simplified manufacturing process of p–n segmented inorganic TE string. Reproduced with permission.^[^ ^167^ ^]^ Copyright 2022, Wiley.

Due to the good processibility of carbon nanocrystals and polymer‐based TE materials, they can be processed into TE yarns/filaments with p–n segmented structure with spinning technologies. Yang and Zhang developed an alternative wet‐spinning strategy to continuously spin SWCNTs‐based p–n segmented TE filaments with good TE properties at scale (**Figure** **9**a).^[^ ^204^ ^]^ However, they still found that the TE properties of p–n segmented filaments were lower than that of homogenous p‐type or n‐type filaments because of the infiltration of low‐viscosity n‐type polyethyleneimine (PEI) dopant with high mobility into p‐type fiber segments during alternate extrusion. In gel spinning, spinning solution usually has high viscosity than wet spinning. It will rapidly lose its mobility during the heat exchange in coagulation bath. Hence, it is suitable to fabricate the TE yarns/filaments with p–n segmented structure. Ding et al. fabricated p–n segmented TE filaments consisting of CNTs and PVA hydrocolloids through a continuous alternating gel‐spinning process (Figure 9b).^[^ ^188^ ^]^ The interface between p‐type and n‐type segments was relatively clear because of the gelation of spinning dopes. Meanwhile, TE filaments with appropriate strength can be further woven into TETs. However, the use of insulating polymers dramatically deteriorates the electrical transport in filaments, leading to low *σ* of 10^−3^ to 10^−1^ S cm^−1^.

The accurate size control of n‐type and p‐type segments is of key importance to the subsequent manufacture of p–n segmented TE yarns/filaments. Especially, in 3D TETs, the length of fiber segments should match the undulated height of the warp strand (or weft strand) to ensure that the n‐type and p‐type segments precisely alternate between hot and cold side. In this regard, coating methods seem to be more controllable and simpler than other spinning methods. Zheng et al. fabricated CNTs‐based segmented TE yarn with PEDOT:PSS (p‐type) and (n‐type) via dip coating with high fabrication efficiency.^[^ ^206^ ^]^

Alternatively, spray printing is another common method for fast preparing wearable electronics with delicate structure, which has been adopted to fabricate p–n segmented TE fibers. In this method, TE inks can be transferred to the substrate via compressed gas flow. Because of the adjustable distance between spray nozzle and target substrate, spray printing can print TE materials on nonsmooth substrates and maintain high resolution of TE patterns.^[^ ^207^ ^]^ Wang et al. prepared integrated fiber‐shaped TE generators by rolling a patterned CNT film and a cellulose nanofiber (CNF) membrane.^[^ ^205^ ^]^ The p‐type and n‐type segments were printed on CNT grid prepared by laser cutting with the assistance of shadow mask (Figure 9c). CNFs were then deposited on obtained grid as insulating layer through electrospinning.

Lastly, owing to the high TE performance of inorganic semiconductors, developing flexible inorganic‐based segmented TE yarns has been preliminarily investigated in recent years. The aforementioned thermal drawing method is still challenging to fabricate p–n segmented TE filaments structure. Recently, Zheng et al. reported a facile routine to produce inorganic‐based segmented TE yarns at scale with good durability and flexibility by involving mold‐assisted cold press and ultrafast high‐temperature sintering process (Figure 9e).^[^ ^167^ ^]^

## 5 Thermal Design of TETs

### 5.1 Thermal Circuit for TETs

In most of the days, the core temperature of human body (≈37 °C) is higher than the environment temperature. The body heat (*Q*) will flow through both skin and TET to air. At present, the heat conversion efficiency is typically 0.1–0.5% for wearable TE devices with the state‐of‐the‐art TE materials.^[^ ^208^ ^]^ According to Equation (8), the stable and high temperature difference (Δ*T*_HC_) between the human body (*T*_H_) and environment temperature (*T*_C_) is a key point to further improve conversion efficiency of TETs. Actually, the temperature difference (Δ*T*_hc_) between the hot side (*T*_h_) and cold side (*T*_c_) of TET is much smaller than Δ*T*_HC_, which is essentially ascribed to the mismatched thermal contact resistance between human body, TET, and environment. The temperature difference utilization ratio (*Φ*) of TETs can be estimated as^[^ ^209^ ^]^

(10) $$\Phi =\frac{\Delta T_{\mathrm{hc}}}{\Delta T_{\mathrm{HC}}}=\frac{\theta_{\mathrm{TET}}}{\theta_{H}+\theta_{\mathrm{TET}}+\theta_{C}}$$

where *θ*_TET_, *θ*_H_, and *θ*_C_ are the thermal resistances of TETs, hot side, and cold side, respectively. Hence, increasing *θ*_TET_ or reducing *θ*_H_/*θ*_C_ is crucial to improve Δ*T*_hc_.

**Figure 10:**
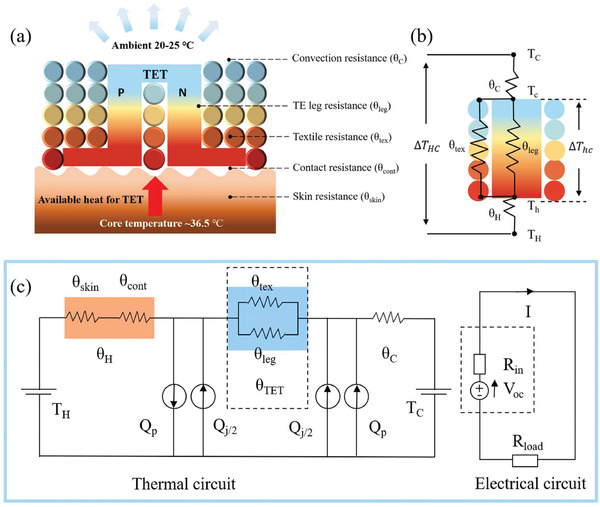
a) Schematic of TET on the human body without heatsink. Circular pattern represents the cross‐section of textile fibers. b) Thermal resistance model of TET in power generation mode, neglecting the Joule heat and Peltier effect. c) Equivalent thermal circuit taking the Joule heat and Peltier effect into consideration. The electrical circuit is shown on the right side in (c).

**Figure** **10** demonstrates a diagram of TET on skin (Figure 10a) and the thermal and electrical circuit of TETs in wearable conditions (Figure 10b,c). *θ*_TET_ is the effective total thermal resistance of TET, which is comprised of the thermal resistance of TE segments (*θ*_leg_), thermal resistance of textiles around the TE legs (*θ*_tex_), and thermal resistance of trapped air in device (Figure 10b).^[^ ^15^ ^]^ *θ*_TET_ can be optimized by manipulating geometric configuration and materials of TE legs and textiles structure, respectively.^[^ ^21^ ^]^ *θ*_C_ is used to evaluate heat exchange between air and TET, which is primarily affected by the surface area available for heat convection.^[^ ^97^ ^]^ *θ*_H_ includes the skin thermal resistance (*θ*_skin_) and the contact thermal resistance (*θ*_cont_) between TETs and skin. *θ*_skin_ is dependent on the device location on the human body, environment temperature, and human activities.^[^ ^210^ ^]^ Compared with traditional TE devices, *θ*_cont_ in TETs is more complicated as it is applied to harvesting body heat energy from curved body surface. The contact interface between TET and rough skin tissue is not standard flat. Especially, those braided 3D textile structures have much smaller contact area in compared to film‐based TE device in practical wearing scenarios, leading to much larger *θ*_cont_. *θ*_cont_ between the skin and TET can be estimated as^[^ ^211^ ^]^

(11) $$\theta_{\mathrm{cont}}=\frac{\left(\lambda /m\right)}{1.25\cdot B\cdot k_{m}\cdot {\left(P/H_{C}\right)}^{0.95}}$$

where *λ* represents the root mean square (RMS) of surface roughness and *m* is the average surface asperity slope, *B* is the macroscopic contact area, and *k*_m_ is the harmonic mean thermal conductivity. *P* is the applied pressure and *H*_C_ is the microhardness of TET. It can be seen that *θ*_cont_ is principally related to the roughness, thermal conductivity, and microhardness of TET. Based on Equation (10), low thermal conductivity of TET will directly reduce *Φ*. Hence, *θ*_cont_ is usually modified via decreasing the roughness and microhardness of TET, such as changing fabric structure and using fine yarns.

Moreover, as electrical current (*I*) flows through TET, Joule heating (*Q*_j_) will be occurred inside TE segments and electrodes, which is modeled by two current sources (*Q*_j_/2) on top and bottom of TET (Figure 10c). The TE segments with certain electrical resistance contributes to a primary part of Joule heating (*Q*_j_ = *I* ^2^ *R*), where *R* is the total electrical resistance of TE segments.^[^ ^97^ ^]^ Joule heating will reduce Δ*T*_hc_. At the same time, Δ*T*_hc_ can be also affected by the Peltier effect. Either cooling or heating at the junction arose from Peltier effect can be calculated by *Q*_P_ = Δ*STI*, where Δ*S* is the Seebeck coefficient difference between TE segments and electrodes, and *T* is the temperature at the junction of TE segments (*T*_c_ or *T*_h_).^[^ ^208^ ^]^ From Figure 10c, it can be seen that Peltier effect will heat the cold side but cool the hot side on TET. Recently, some reports mentioned that the Joule heating can be neglected during the thermal design of TETs because of its limited temperature drop on TETs, whereas the Peltier effect cannot be ignored in general unless the thermal conductivity of TE legs (*k*_leg_) is far more less than that of textile‐based filler (*k*_tex_).^[^ ^17^ ^]^

### 5.2 Parameters Optimization of TETs

As aforementioned, Δ*T*_hc_ of TETs is restricted by large extrinsic thermal parasitic resistances (*θ*_H_ and *θ*_C_). For example, Δ*T*_hc_ on wearable devices is typically ≈0.5–3 K as the temperature difference between body and ambience is ≈10–15 K.^[^ ^97^ ^]^ The general routine to improve Δ*T*_hc_ is to decrease both *θ*_H_ and *θ*_C_ while increase the thermal resistance of TET for the thermal matching between body, TETs, and ambience.

In order to reduce *θ*_C_, heat sinks in different configurations and types can be introduced to dissipate extra heat on the top of TE device to environment. Rigid, heavy, and bulky heat sinks are inconvenient during human activities in daily life, and thus wearable devices commonly chose fin‐shaped or pin‐shaped metal heat sinks,^[^ ^212^ ^]^ whereas it is still unpleasant to wear them in large area.^[^ ^213^ ^]^ Therefore, it is crucial to develop novel heat sink that is efficient, flat, and flexible. Recently, some neoteric materials (i.e., foam metal,^[^ ^214^ ^]^ aqueous polymer,^[^ ^215^ ^]^ phase change materials,^[^ ^216^ ^]^ radiative‐cooled materials,^[^ ^217^ ^]^ etc.) have been used to enhance the heat exchange capacity of TEGs but there is limited work in combination with TETs. In addition, Xu et al. developed a multifunctional mushroom‐like cooper electrode to achieve heat concentration and dissipation simultaneously which can help to fabricate high‐performance TETs without introducing an external heat sink.^[^ ^218^ ^]^

As for *θ*_H_, it is imposed by the skin condition (*θ*_skin_) and interface condition (*θ*_cont_) between skin and TET. *θ*_skin_ is a complicated function of specific physiology and the *θ*_skin_ of TET is usually modified by changing TET location on body. According to the formulation of *θ*_cont_, increasing the contact area, decreasing roughness of TET, or increasing pressure can be utilized to reduce *θ*_cont_. Suarez et al. studied the relationship between different contact pressure (0–1 kPa) for various locations on the body and heat transfer coefficient of the rigid TEG/skin interface.^[^ ^17^ ^]^ For reference, 0.5 and 0.8 kPa are comparable to tight clothing. They found forearm is a good location for TEG where TEG achieved the largest heat transfer coefficient and its heat transfer coefficient ranges from 50 to 70 W m^−2^ K^−1^ for tight fitting clothing. This study demonstrated the necessity of developing stretchable TETs which can apply additional pressure and enlarge contact surface between devices and skin. Meanwhile, due to its flexibility, TET theoretically outperforms above rigid counterparts. Depositing materials with high thermal conductivity on the hot side of TEGs is also an effective way to decrease *θ*_H_. Wang et al. observed that Δ*T*_hc_ approximately approached maximum value and varied little when thermal conductivity of coating layer is larger than 10 W m^−1^ K^−1^ based on finite element analysis.^[^ ^211^ ^]^

Besides controlling thermal resistances of external conditions which is typically challenging, TETs should possess highly controllable thermal resistance to achieve good thermal match to the external environments in order to enhance Δ*T*_hc_ across TETs toward high output thermoelectric power.^[^ ^14^, ^31^ ^]^ Due to the coupling of the thermal circuit and electric circuit created by Peltier and Joule effect, co‐optimizing the thermal and electric resistance of TETs should be taken into consideration simultaneously. Under the maximum output power conditions, heat conduction plays the dominant role in heat harvesting over terminal effects of Peltier and Joule terms. Ignoring Peltier and Joule effects, thermal and electric matching conditions have been shown as follow: *θ*_TET_ = *θ*_H_ + *θ*_C_ and *R*_TET_ = *R*_load_.^[^ ^219^ ^]^ In case of thermal circuit with Peltier and Joule effects (Figure 10c), the thermal and electric matching conditions could be expressed as follow: $\theta_{\mathrm{TET}}=\left(\theta_{H}+\theta_{C}\right)\;\sqrt{ZT+1}$ and $R_{\mathrm{TET}}=R_{\mathrm{load}}\;\sqrt{ZT+1}$.^[^ ^220^ ^]^

In the design of traditional TE devices, *θ*_TET_ and *R*_TET_ can be simply optimized via changing the height‐to‐area ratio of TE legs and fill factor,^[^ ^97^ ^]^ which is also applicable to the thermal optimization of TET. It has been proposed that large height‐to‐area ratio contributes to improve *θ*_TET_. Nevertheless, there is a limit to the size of TE legs that can be prepared by existing methods^[^ ^17^ ^]^ and the flexibility of TETs will be reduced with the TE leg height (TET thickness) increasing. In general, the value of *k*_leg_ is higher than that of *k*_tex_, so reducing the number of TE legs (fill factor) for a given TE device can increase *θ*_TET_ but the output voltage of TE device is proportional to the number of TE legs. Therefore, based on thermal and electrical matching, finding ideal height‐to‐area ratio and fill factor of TE legs are key aspects to design TETs with high output performance. The theoretical number of TE legs (*N*) can be estimated by^[^ ^221^ ^]^

(12) $$N_{\mathrm{ideal}}=\sqrt{\frac{R_{\mathrm{load}}}{\left(\theta_{H}+\theta_{c}\right)}\frac{\left(\sigma_{n}+\sigma_{p}\right)}{\left(k_{n}+k_{p}\right)}}$$

where *σ*_n_ and *σ*_p_ are electrical conductivity of p‐type and n‐type TE legs; *k*_n_ and *k*_p_ are thermal conductivity of p‐type and n‐type TE legs. The optimized height‐to‐area ratio (*l/A*)_ideal_ can be estimated by

(13) $$\begin{array}{ccc} {\left(l/A\right)}_{\mathrm{ideal}} & = & \frac{R_{\mathrm{load}}}{N_{\mathrm{ideal}}}\left(\sigma_{n}+\sigma_{p}\right)\\ & = & \sqrt{R_{\mathrm{load}}\left(\theta_{H}+\theta_{c}\right)\left(k_{n}+k_{p}\right)\left(\sigma_{n}+\sigma_{p}\right)} \end{array}$$

**Figure 11:**
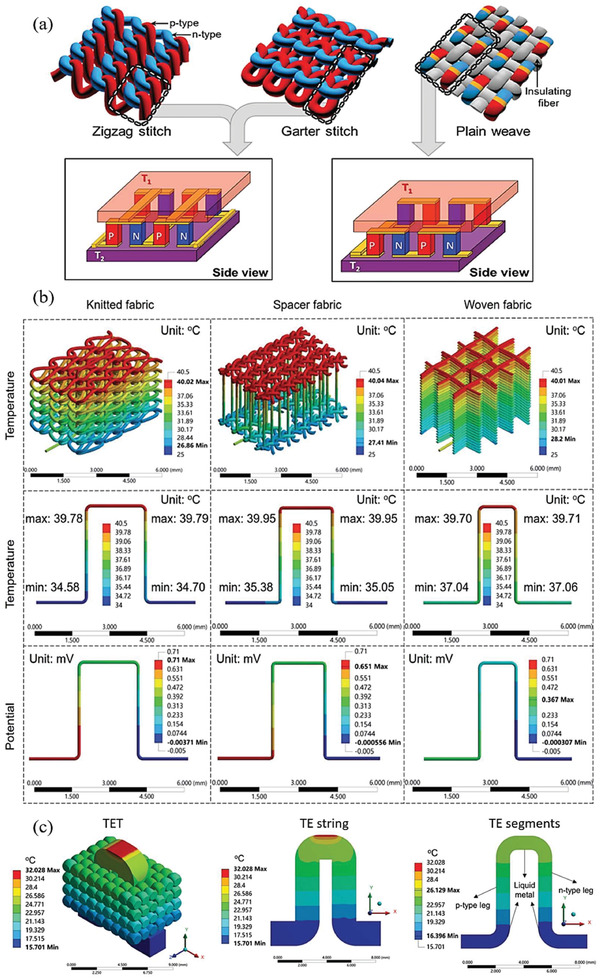
a) Illustration of TETs with zigzag‐stitch, garter‐stitch, or plain‐weave structures, respectively. Reproduced with permission.^[^ ^223^ ^]^ Copyright 2016, Wiley. b) Finite element simulations of TETs utilizing weft‐knitted, warp‐knitted spacer, and woven fabrics at a specific fabric weight, thickness, and fixed hot‐side temperature (40 °C).^[^ ^206^ ^]^ c) Temperature distribution of the TET, PDMS layer, and TE segments. Reproduced with permission.^[^ ^167^ ^]^ Copyright 2022, Wiley.

Except for above optimizing methods, which is served as the universal strategy for wearable TEGs, TETs usually involve more structural parameters that can be manipulated, due to their more complex structure compared to traditional rigid or wearable TE devices. It gives us more opportunities to control the thermal performance of wearable TEG. For example, we can alter the thermal resistance of TET by manipulating the geometry and thermal properties of TE filaments, the selection of textile fibers with different geometries and thermal properties as well as the arrangement and volume of textile fibers in TET using different textile processes, such as weaving, knitting, knotting, etc. For example, Lee et al.^[^ ^222^ ^]^ studied TE performance of TETs with zigzag‐stitch, garter‐stitch, or plain‐weave structures. They found that the TET with TE yarns electrically connected in series (plain‐weave textile) gained higher output power than that connected in parallel (zigzag‐stitch and garter‐stitch) as shown in **Figure** **11**a. And the output properties of TETs can be further increased by increasing the diameter of warp yarns (textile thickness) and the density of TE couples and TE yarns, since they can help to achieve high Δ*T*_hc_ and obtain large fill factor, respectively. Owning to the relatively limited knowledge about the influence of textile structure on TETs, Zheng et al. in the year of 2020 shed light on the textile structure effect on the temperature distribution in TETs with the combination of experimental characterization and finite element simulations (Figure 11b).^[^ ^206^ ^]^ They found that Δ*T*_hc_ applied on TE segments in spacer and knitted fabrics consisted of irregular yarn arrangement is much larger than that in plain woven fabrics for a given thickness of TET, because spacer and knitted fabrics with good thermal insulation capability (large *θ*_tex_) can trap more static low‐thermal‐conductivity air^[^ ^208^ ^]^ and thus reduce heat transfer from hot side to cold side of TET. Recently, they studied the temperature distribution of the inorganic TET, woven from a TE strings configuration with TE couples on PI filaments encapsulated with a polydimethylsiloxane (PDMS) elastomer, and explored the effect of PDMS insulator on actual Δ*T*_hc_ across TE segments (Figure 11c).^[^ ^167^ ^]^

### 5.3 Structure Categories of TETs

**Figure 12:**
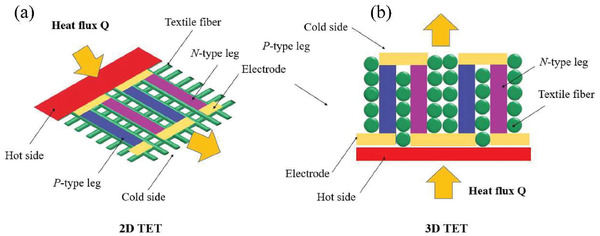
Schematic overview of a) 2D TET and b) 3D TET.

As aforementioned, TETs can be mainly categorized into 2D and 3D TETs. For 2D TETs, fiber‐shaped TE segments are perpendicular to the thickness of TETs, which can solely harvest thermal energy in the in‐plane direction (**Figure** **12**a). However, the main direction of heat dissipation from skin is to the thickness direction of fabrics. Hence, it is not efficient to directly use 2D TETs to harvest waste heat from human body.

On the other hand, the fiber‐shaped TE segments in 3D TETs arrange in the thickness of fabrics and thus 3D TETs can efficiently utilize the heat dissipated in the out‐of‐plane direction of TETs (Figure 12b). Previous attempts proposed that TETs utilizing heat in the thickness direction (3D TET) gained higher output power than that in the plane of textile (2D TET), owing to the latter with larger Δ*T*_hc_.^[^ ^223^, ^224^, ^225^ ^]^ 3D TETs usually display high integration density compared to 2D TETs, facilitating to obtain excellent power density. Meanwhile, 3D TETs generally show higher flexibility and durability than 2D TETs because fiber‐shaped TE segments can be protected by surrounding textile fibers and bear limited deformation during 3D deformation. Hence, 3D TETs are promising to other types and will be the main research focus in the development of TETs.

## 6 Manufacture of TETs

### 6.1 Fabrication of 2D TETs

**Figure 13:**
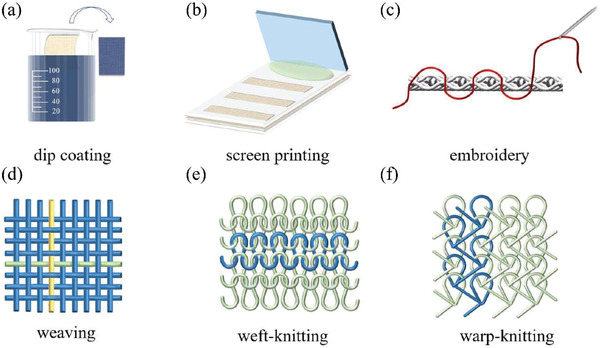
The illustration of the manufacturing process of TETs.

Common fabrication methods for 2D TETs mainly include screen printing and coating. Compared with dip coating, drop casting has been wildly used to fabricate functional fabrics with large volume (**Figure** **13**a). The main fabrication principle and process for these methods are the same as that for fabricating TE yarns/filaments and thus we will not discuss any further in this section.

*Screen printing* is a popular technique in textiles processing (Figure 13b). Recently, it has been widely used in electronics application due to its simple process and university for various substrates and inks. Viscous inks containing TE materials are forced through stencils/patterned mesh by a squeegee‐type device to obtain patterned TE textiles.

Organic or inorganic TE materials all can be processed into TE inks. The viscosity is an essential index to evaluate the processibility of ink in screen‐printing process which decides the deposition resolution and quality of TE coating. For organic TE materials with good dispersibility in solution, the viscosity can be adjusted by changing concentration of polymers. However, inorganic TE materials cannot be directly dispersed into solution and the viscosity of inks depends on the viscosity of the solvent. The key to fabricate inorganic‐based TETs via screen printing is to find appropriate additives to improve the dispersibility of inorganic TE materials and obtain ideal ink viscosity. In early studies, some insulating polymers such as polyvinylidene fluoride (PVDF) and PVA are chosen as binders.^[^ ^226^ ^]^ However, for this kind of coating, TE materials will be encapsulated by insulating polymers to form aggregates, leading to poor *σ*.^[^ ^227^, ^228^ ^]^ In order to improve the *σ* of the printed film, insulating binders are replaced by conducting polymers such as PEDOT:PSS, whereas the *S* of films will be reduced due to the low *S* of conducting polymers.^[^ ^229^ ^]^ Recently, CNF is extensively used in screen‐printing process. A small quantity of CNF (0.45–0.60 wt%) can achieve suitable printing viscosity and it can be decomposed in sintering process of inorganic materials.^[^ ^230^ ^]^ Another challenge is that fabrics usually have high porosity and roughness because of the unique yarn interweaving structure, which will break the continuity of TE coating and result in the low *σ* of TETs, especially for the TE coating with thin thickness. Shin et al. solved this problem via pre‐printing chitosan layer before printing TE materials.^[^ ^231^ ^]^

### 6.2 Fabrication of 3D TETs

Weaving, knitting, and embroidering are all traditional manufacturing techniques of textiles, which can be applied to process TE yarns/filaments into 3D TETs.^[^ ^232^ ^]^ Compared with weaving and knitting, embroidery can easily control the arrangement of TE legs in the fabric by needles, without destroying textile substrates and thus it has been widely used to fabricate 3D TETs. The embroidery process has been shown as follow: the needle with TE yarns/filaments pierces the fabric held in a frame from top, then moves to the defined position and finally returns to the top of fabric (Figure 13c). Various commercialized fabrics (i.e., woven fabrics, knitted fabric, nonwoven fabrics, etc.) and different TE filaments and yarns are ideal materials used for developing 3D TETs via embroidery. Base fabric in TETs can provide a support to TE filaments/yarns so 3D TETs produced by embroidery usually exhibit good mechanical deformability.

*Weaving* is one of the most important technologies in the textile industry, which is the interlacement of lengthwise yarn (warp yarns) and crosswise yarn (weft yarn) at a right angle (Figure 13d).^[^ ^233^ ^]^ In weaving process, warp yarns usually keep straight under the influence of tension and weft yarns are woven through the warp yarns (above and below) based on different rules. Generally, 1D TE yarns/filaments are used as weft yarn because warp yarns always need to withstand the additional tension and repeated frictions from weaving machines during processing procedure. In woven fabric, warp yarns and weft yarns are crossed and interwoven to provide a stable mechanical structure to 3D TETs. The thermal properties of woven fabrics are greatly dependent on the weave and linear density of weft yarns.^[^ ^234^ ^]^ For instance, plain‐woven fabrics usually display higher thermal conductivity than twill and honeycomb weave, due to their lower porosity and thickness as well as regular yarn arrangement.^[^ ^235^ ^]^ The *θ*_tex_ of woven TETs can be achieved via changing fabric structures and tuning weave parameters.

*Knitting* is an effective way to develop stretchable 3D TETs with complex structure, which is mainly divided into weft‐knitted and warp‐knitted textiles. For weft‐knitted fabric, yarns are curled into loops and then loops are intermeshed along the width of the fabric (Figure 13e), while for warp‐knitted textiles, loops are made from different yarns in a vertical way along the length of the fabric (Figure 13f). Unlike warp‐knitted textiles, weft‐knitted fabrics have high extension at low tension, excellent stretchability as well as voluminous structure which is beneficial to reduce the *θ*_TET_ of TETs according to theoretical analysis and improve the comfort of TETs simultaneously. Warp‐knitted structure is rather changeable and thus it is able to develop TETs with complex structure.

The strength of TE filaments/yarns decides the feasibility of high‐volume production of TETs using industrial machinery. Cotton yarns, as a kind of common commercial yarns are usually woven, knitted, or embroidered into fabric in textile industry and its tensile strength is generally higher than 10 cN tex^−1^. Therefore, if the breaking strength of TE filaments/yarns is comparable to that of cotton yarns, it is possible to achieve the continuous production of TETs via textile machine in a large scale. For instance, we assume that a density of conducting polymer is about 1 g cm^−3^ and thus the tensile strength of conducting polymer‐based TE filaments/yarns should achieve 9800 MPa, which is far greater than most of existing values of TE filaments/yarns (Figure 8). Most of tensile strength reported previously is for TE monofilaments and the tensile strength can be further improved by twisting TE filaments into TE multifilament via two rolls.^[^ ^40^ ^]^

## 7 Thermoelectric Power Generation of TETs

### 7.1 2D TETs

**Figure 14:**
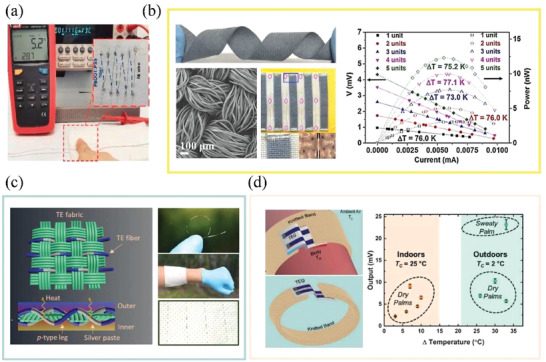
a) The output voltage of 2D TET when one end of the 2D TET was touched by a fingertip. Reproduced with permission.^[^ ^178^ ^]^ Copyright 2020, Elsevier. b) SEM image and photograph of PEDOT:PSS‐treated polyester fabric (left), and output properties of PEDOT:PSS‐based 2D TET (right). Reproduced with permission.^[^ ^236^ ^]^ Copyright 2015, Springer Nature. c) Schematic of 2D TET and digital photos of PEDOT:PSS/Te NWs filaments, fabric, and wearing demonstration. Reproduced with permission.^[^ ^183^ ^]^ Copyright 2020, American Chemical Society. d) Illustration of a wearable TET design and corresponding output voltage as wearing on palm in different conditions. Reproduced with permission.^[^ ^238^ ^]^ Copyright 2019, Wiley.

2D TETs have planar construction leading to mainly harvest energy in in‐plane direction. 2D TETs can be fabricated by fixing n‐type and p‐type TE yarns/filaments or TE strips on the surface of fabrics via pasting or sewing and subsequently n‐type legs and p‐type legs are connected by electrodes in series. In general, most of the commercial fabrics can be used as substrates of 2D TETs, such as cotton, silk, chemical fiber‐based woven, knitted, and nonwoven fabrics. Wen et al. developed a 2D TET via sewing five pairs of p‐type PEDOT:PSS fibers and n‐type Ni wires onto a piece of cloth.^[^ ^178^ ^]^ This 2D TET delivered a high output voltage ≈1.11 mV upon a fingertip touch at one end of 1D TETs (**Figure** **14**a) and its output power density achieved ≈0.273 µW cm^−2^. Du et al. prepared TE strips by immersing commercial polyester fabrics into PEDOT:PSS/DMSO mixture and then they mounted some TE strips to untreated polyester fabrics using silver paint (Figure 14b).^[^ ^236^ ^]^ The output voltage and maximum output power of 2D TETs were only 4.3 mV and 12.29 nW at *∆T* ∼ 75.2 K, respectively, due to the poor TE performance of TE strips (the maximum PF of 0.045 µW m^−1^ K^−2^) and the lack of n‐type TE legs. When polyester fabrics were replaced with cotton fabrics and Constantan wires were introduced as n‐type TE legs, the PF of PEDOT:PSS fabrics was improved to 212.6 nW at *∆T* = 74.3 K.^[^ ^237^ ^]^

When 2D TETs are directly used to harvest the heat in the out‐of‐plane direction, heat conversion efficiency is extremely low. Xu et al. assembled a 2D TET by embroidering 28 pairs of PEDOT:PSS/Te NWs filaments with high TE properties into a commercial textile (Figure 14c).^[^ ^183^ ^]^ Nevertheless, the output voltage and output power of 2D TETs were only 25.9 mV and 197.9 nW even at a given *∆T* ∼ 41 K because *∆T* across TETs was much less than given value as demonstrated by finite‐element analysis and simulation.

Inorganic TE materials can be made into 2D TETs by screen printing. For example, Cao et al. printed p‐type Sb_2_Te_3_ and n‐type Bi_1.8_Te_3.2_ on high‐temperature glass fiber textile,^[^ ^239^ ^]^ containing eight pairs of thermocouples, generated an output voltage of 26.6 mV and maximum output power of 2304 nW at *∆T* = 20 K. Though inorganic TETs exhibit higher output properties, the flexibility of inorganic TE coating and the adhesion to textile are lower than that of organic materials.

It has been a conundrum for 2D TETs to efficiently utilize the temperature difference between skin and environment. There is an optional way to partially address this issue by appropriate architecture design to convert planar structure of 2D TETs to vertical configuration. Allison and Andrew prepared a 2D TETs via vapor printing PEDOT:Cl onto commercial cotton fabrics.^[^ ^238^ ^]^ Interestingly, the output voltage of 2D TETs achieved as high as 23 mV on hand, by integrating 2D TET into a specially designed wool band. The wool band with heavy thickness kept stable *∆T* across TET over time (Figure 14d).

### 7.2 3D TETs

In 3D TETs, n‐type and p‐type TE segments are embedded in fabrics and then TE legs are combined by electrodes on the surface of fabrics. Because of the arrangement of TE legs along the thickness direction of the textile, deformation mostly takes place on electrodes when 3D TETs are bent, twisted, or stretched. Hence, either rigid inorganic or flexible organic TE materials can be used to fabricate 3D TETs with excellent flexibility by waving, embroidering, screen printing, or other unique technologies.

**Figure 15:**
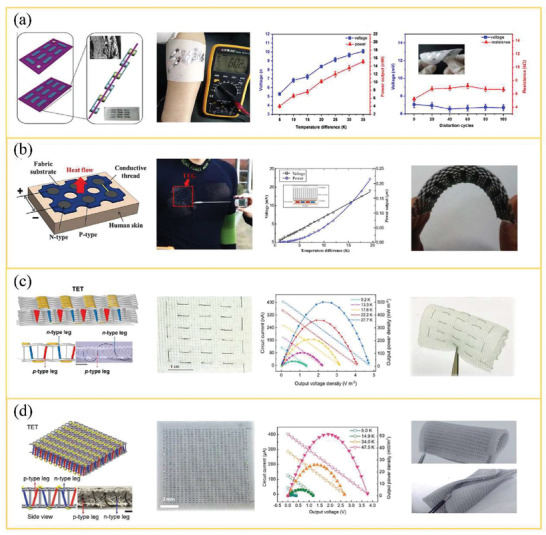
a) Schematic illustration of a silk‐based 3D TETs composed of Bi_2_Te_3_ and Sb_2_Te_3_; output voltage of a silk‐based TET attached to the arm at 20 °C; output properties of a silk‐based TET; effects of twisting on the internal output voltage and internal resistance of the silk‐based TET. Reproduced with permission.^[^ ^240^ ^]^ Copyright 2016, Elsevier. b) Diagram of polymer‐based 3D TETs composed of Bi_0.5_Sb_1.5_Te_3_ and Bi_2_Se_0.3_Te_2.7_; photograph of the polymer‐based TET embedded in the shirt for clothing application; output properties of 12‐couple TET concerning temperature difference; image of bending the polymer‐based TET by hand. Reproduced with permission.^[^ ^241^ ^]^ Copyright 2014, Institute of Physics. c) Schematic illustration of the spacer fabric‐based 3D TETs fabricated with SWCNT‐based p–n segmented TE filaments; photograph of a spacer fabric‐based TET; short‐circuit current and output power density as a function of output voltage density at different Δ*T*; image of folding the spacer fabric‐based TET. Reproduced with permission.^[^ ^204^ ^]^ Copyright 2022, American Chemical Society. d) Diagram of the spacer fabric‐shaped 3D TETs fabricated by sewing CNT‐based p–n segmented TE yarns; photograph of a spacer fabric‐shaped TET; circuit current and power density versus voltage at various Δ*T*.^[^ ^206^ ^]^

Screen printing can also be used to effectively fabricate organic/inorganic 3D TETs. Different from the manufacturing process of 2D TETs, periodic aligned holes should be burned out in 3D fabric according to pre‐designed size and arrangement, before printing TE inks. Lu et al. developed silk‐based 3D TETs consisting of nanostructure Bi_2_Te_3_ and Sb_2_Te_3_ via screen printing (**Figure** **15**a).^[^ ^240^ ^]^ The maximum voltage and power outputs of this device with 12 pairs of thermocouples were ≈10 mV and ≈15 nW at *∆T* ∼ 27.7 K, respectively. Owing to the good flexibility of silk fabrics and unique 3D structure of TETs, output properties of this inorganic device had negligible change after 100 cycles of bending and twisting. For further improving the feasibility of 3D TETs in the form of clothing, Kim et al. chose conductive thread as electrodes and sewed them into a polymer‐based textile with specific grid structure (Figure 15b).^[^ ^241^ ^]^ The 3D TET was prepared by depositing Bi_0.5_Sb_1.5_Te_3_ and Bi_2_Se_0.3_Te_2.7_ inks in the textile with conductive thread via screen printing. As worn on human body, the output power was 224 nW at an environment temperature of 5 °C. This device did not have obvious deformation after bending and stretching several times, which demonstrated the feasibility of the TET for applications in real life.

It is well‐known that the annealing process is of great importance to the TE properties of inorganic materials. Hence, polyimide or glass fabrics with excellent thermal stability are more suitable to be used as substrate in inorganic‐based TETs. Sun et al. obtained high‐performance 3D TETs via introducing annealing process and modifying the connection between TE legs and electrodes.^[^ ^242^ ^]^ When this TET was worn on human wrist, it showed not only outstanding flexibility but also achieved an output power of up to 3 µW at an ambient temperature of 15 °C. However, the PDMS coating may impair the wearing comfort of TETs. In addition to using TE materials with high *zT*, the thermoelectric power output can be enhanced through increasing the number of p–n TE couples. Hend et al. developed a type of 3D TET with 16 pairs of p‐type PEDOT:PSS and n‐type poly[Na(NiETT)] segments via a stencil assistant transfer printing technique, yielding a maximum output voltage of 3 mV at *∆T* ∼ 3 K. The output voltage achieved 47 mV at *∆T* = 3 K when 3D TET was assembled with 432 pairs of p–n TE segments.^[^ ^243^ ^]^

In previous attempts, the prepared p–n segmented TE fibers/yarns are usually processed into 3D TETs by embroidering them into thick textiles. Yang and Zhang^[^ ^204^ ^]^ obtained 3D TETs by embroidering SWCNTs‐based p–n segmented TE filaments fabricated via wet‐spinning into the spacer fabric and this device displayed a good output power density of 501 nW m^−2^ at *∆T* ∼ 27.7 K (Figure 15c). Zheng et al. prepared CNTs‐based TE yarns with p–n segmented structure by dip coating and embroidered as‐prepared TE yarns into spacer fabric (Figure 15d).^[^ ^206^ ^]^ The power density of this 3D TET was up to 51.5 mW m^−2^ at a Δ*T* ∼ 47.5 K. For 3D TETs prepared by printing or embroidery, the 3D structure of TETs is mainly depended on the thickness of textile substrate. 3D fabrics with large thickness (<3–5 mm, similar to down jacket) can efficiently thermally insulate warm skin from cold environment. Too large thickness may limit the natural movement and metabolism of body in daily life. Hence, it is still urgent to find a novel routine to develop 3D TETs with outstanding output power and better wearing comfort simultaneously. Directly knitting or weaving TE fibers into 3D TETs is probably a feasible way.

**Figure 16:**
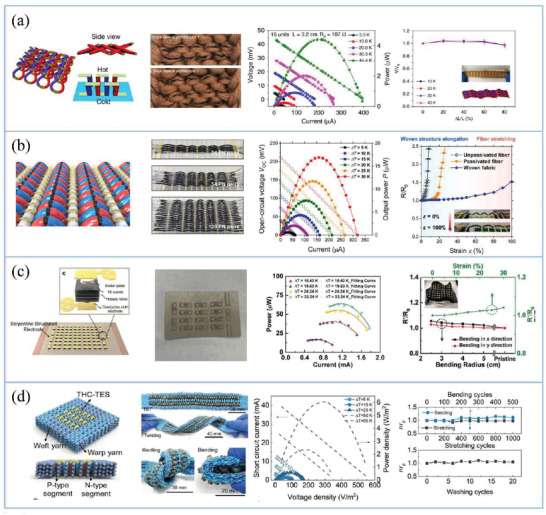
a) Schematic diagram of warp‐knitted TET; the front view and back view of warp‐knitted TET; output properties of warp‐knitted TET with TE yarn; output properties degradation concerning longitudinal strain. Reproduced with permission.^[^ ^224^ ^]^ Copyright 2020, Nature Springer. b) Schematic of TET woven from CNTs/PU TE filaments; process of weaving and electrically passivated CNTs/PU TE filaments; output properties of woven TET with 108 TE couples; internal electrical resistance of woven TET as a function of applied strain. Reproduced with permission.^[^ ^245^ ^]^ Copyright 2022, Elsevier. c) Illustration of elastic fabric‐based TET and configuration for one connected TE cuboid; image of TET; output properties of TET with 48 TE couples; resistance variation (*R’/R*_0_) of TET with 48 TE couples. Reproduced with permission.^[^ ^247^ ^]^ Copyright 2022, Wiley. d) Schematic of TET fabricated by woven bead‐like TE strings; optical images of flexibility; output properties of TET; the internal resistance variation (*r’/r*_0_) of TET after stretching, bending, and washing. Reproduced with permission.^[^ ^167^ ^]^ Copyright 2022, Wiley.

Beside flexibility, stretchability is also important for TETs to meet the adaptability of stretching, torsion, and compression caused by human daily activities and ensure tight contact between the dynamic curved surface of human body and TETs.^[^ ^244^ ^]^ Traditional weft‐knitted fabrics with coil construction exhibit ultrahigh elasticity up to 500%, being much more stretchable than other kinds of fabrics.^[^ ^203^ ^]^ Sun et al. developed 3D TETs with excellent stretchability and output power by utilizing the specific *π*‐type coil structure of knitting fabrics (**Figure** **16**a).^[^ ^224^ ^]^ They prepared p–n segmented CNTs‐based TE fibers via electrospray technology. Then TE fibers wrapped with acrylic fibers were knitted into *π*‐type TE modules. The peak power density of this stretchable TET was 70 mW m^−2^ at *∆T* ∼ 27.7 K due to the high thermal resistance caused by the coil structure of fabrics and loose arrangement of acrylic fibers. It was able to be stretched 80% without output reduction. Similarly, the woven fabrics with *π*‐type coil structure also have stretchability. In order to further improve the stretchability of 3D TETs, stretchable TE yarns/filaments are brought into woven TETs. When the elongation of deformable coil structure can no longer withstand additional strain, the elastic TE yarns/filaments can maintain conductive pathways and limit the reduction of TE performance of TETs. Jang et al. designed wearable TETs with exceptional stretchability up to 100% by combining the inherent stretchability of TE yarns/filaments and deformability of textile structure (Figure 16b).^[^ ^245^ ^]^ They deposited CNTs ink on elastic Pu fibers via drop casting and then obtained elastic p–n segmented TE fibers via dip coating with PE mask. Finally, the PU passivation was encapsulated on the prepared p–n segmented TE fibers to improve the durability. The 3D TETs fabricated by waving TE fibers exhibited the normalized power density of 1.7 × 10^−3^ µW cm^−2^ K^−2^.

It is of key importance to carefully design the architecture of TET to offer structural stretchability without sacrificing high TE performances of inorganic semiconductors. In general, the stretchability of 3D inorganic TETs mainly depends on the mechanical properties of fabric substrates and electrodes. Hence, rigid inorganic TE pillars also can be used as TE legs to assemble stretchable 3D TETs. The stretchable electrodes can be obtained by depositing high‐conductive materials on elastic films or using liquid metal.^[^ ^246^ ^]^ Hou et al. fabricated the deformable by elastic fabric‐based TET with Bi_2_Te_3_‐based alloys cuboids interconnecting with conductive polyester fiber‐based serpentine electrodes (Figure 16c).^[^ ^247^ ^]^ The 3D TET kept excellent TE performance with the peak output power of 64.10 µW and output voltage of 111.49 mV at *∆T* ∼ 33.24 K when strain reached 30% or on arbitrarily shaped heat source. In 2022, Zheng et al. developed mechanically stable TE strings configured by inorganic p‐ and n‐type segments on polyimide filaments with liquid metal as interelectrode and PDMS elastomer as encapsulation (Figure 16d).^[^ ^167^ ^]^ Then, TE strings as weft yarns and elastic yarns as warp yarns were woven into a 3D multilayer TET via a semiautomatic weaving machine, which demonstrated outstanding stretchability (100% elongation), flexibility (bending radius of 2 mm), and washability (>20 washing cycles).^[^ ^167^ ^]^ Meanwhile, this inorganic 3D TET exhibited ultrahigh output power density of 0.58 W m^−2^ at the *∆T* = 25 K.

## 8 Personal Thermoregulation of TETs

Apart from power generation, wearable thermoelectric devices also provide great potentials for cooling the microclimate of the human body based on Peltier effect.^[^ ^248^ ^]^ Compared with some of flexible film‐based coolers, TETs are promising to achieve conformal geometrical contact with human body and effective localized thermoregulation due to their intrinsic flexibility.

**Figure 17:**
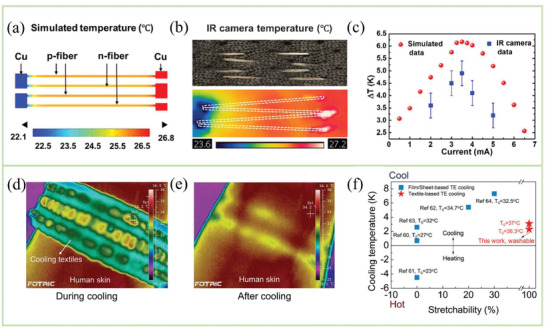
a,b) The finite element modeling simulated and IR actual camera‐captured temperature distribution. Reproduced with permission.^[^ ^191^ ^]^ Copyright 2016, Wiley‐VCH. c) Simulated and IR‐measured cooling temperature versus input electrical current. Reproduced with permission.^[^ ^191^ ^]^ Copyright 2016, Wiley‐VCH. d,e) The skin temperature is 34.2 °C before cooling.^[^ ^167^ ^]^ After cooling, the skin temperature is reduced to 32.3 °C. Copyright 2022, Wiley. f) The comparison of cooling effect of 3D TECs with film/sheet‐based TE cooling. Reproduced with permission.^[^ ^167^ ^]^ Copyright 2022, Wiley.

Until now, there are limited reports on organic cooling applications since they usually show negligible Peltier effect because of their obvious Joule heating caused by their relatively poor *σ*.^[^ ^249^ ^]^ Hence, attempts about wearable thermoelectric coolers mainly focus on inorganic TE materials. It is a challenge to construct inorganic TE materials with a rigid and brittle nature into TE filaments/yarns for weaving textile‐based thermoelectric coolers (TECs). Zhang et al. developed 2D TECs by weaving flexible crystalline p‐type Bi_0.5_Sb_1.5_Te_3_ and n‐type Bi_2_Se_3_ filaments via thermal drawing into a fabric and this cooler displayed a maximum cooling temperature of 4.9 K at the current of 3.5 mA (**Figure** **17**a–c).^[^ ^191^ ^]^ In 2022, Zheng et al. fabricated stretchable 3D multilayer TECs using the bead‐like ternary hierarchically coaxial Bi‐Te compounds‐based TE strings as weft yarns, which for the first time cools the skin temperature by a drop of 3.1 K (Figure 17d–f).^[^ ^167^ ^]^

## 9 Cost Metrics of TETs

While energy conversion efficiency is an important metric, the cost of generating electricity is also crucial to the commercialization of TEGs. Some attempts devoted to develop high‐performance wearable TEGs via organic/inorganic hybridization, physical vapor deposition on flexible substrate, nanostructing, and so on.^[^ ^63^, ^250^ ^]^ However, the increase of capital cost should be taken into account to determine the net effect on the TEGs costs and the breakthrough in the TEGs performance.^[^ ^251^ ^]^ The total cost of a typical TEGs (*C*[*$*]) can be estimated as follows^[^ ^252^ ^]^

(14) $$C\left[\$\right]=\left(C^{\prime \prime \prime }H+C^{\prime \prime }\right)\;\left(A_{P}+A_{N}\right)$$

where *C*‴ and *C*″ are the volumetric cost and area cost, respectively, *A*_P_ and *A*_N_ are cross‐sectional area of p‐type and n‐type TE legs; *H* is the height of TE legs. *C*‴ is comprised of the cost of TE materials, manufacturing costs (e.g., hot pressing and ball milling) and other costs that scale with TE materials. *C*″ includes the cost of metallization, areal manufacturing costs (e.g., dicing and cutting), and another costs that scale with the area of the module.^[^ ^253^ ^]^

In most of TETs, the cross‐sectional area of TE legs, especially fiber‐based TETs, is smaller than that of bulk inorganic semiconductor‐based TEGs, which can effectively reduce cost via decreasing *A*_P_ and *A*_N_. In addition, TE legs of TETs can be fabricated via spinning, coating, or printing, leading to the decreased energy consumption caused by high‐temperature manufacturing methods. And p–n segmented TE yarns/filaments can be directly integrated into a textile, which avoids cutting and connecting process and provides a convenient and less costly way to achieve heat harvesting. Meanwhile, raw materials (e.g., wool, cotton, and polyester) of textiles are cheap and textile manufacturing techniques are mature. Hence, compared with other kinds of wearable TEGs, TETs show great promise in cost reduction.

## 10 Summary and Perspectives

In this review, we have comprehensively reviewed the research progress in textile‐based TE devices, intending to deliver insights on TETs from the aspects of the selection and optimization of TE materials, their interfacial properties with textile fibers as well as configuration design and scalable fabrication of TETs. It is still a long road to develop truly wearable TET with high thermoelectric, mechanical, and wearing performances. Toward this utmost goal, there are some aspects that should be paid with more attentions and efforts.

1) Developing organic thermoelectric materials with *zT* > 1. Especially, enhanced thermoelectric performance of conducting polymers is highly demanded because they can meet almost all requirements for TET fabrication except their low TE performance. The thermoelectric transport theory for organic thermoelectric materials is still far from well developed, which cannot accurately guide the design and synthesis of new organic thermoelectric materials.
2) Adopting inorganic semiconductors to fabricate flexible TE filaments with high TE and mechanical properties at scale. There are many material options if inorganic semiconductors can be designed to be flexible TE filaments. Recently, the thermal drawing method and some textile manufacturing processes (i.e., dry spinning) show great potential to address this issue. Additionally, the interaction between TE materials and textile fibers has been overlooked during the design of TE fibers and devices, which is significant to improve the mechanical durability of TETs.
3) Find the novel routines to prepare p–n segmented TE fibers/yarns with high TE properties in a large scale. At present, there are still some problems in current methods such as complex process, poor controllability for the size of TE segments, etc.
4) Optimizing the thermal design of TET to further enhance the temperature difference utilization. Due to the large external parasitic thermal resistance of TETs, it is difficult to achieve thermal matching between TETs and parasitic resistance in realistic fabrication process. It might be a feasible routine to design TET with complex textile structure to fulfill the requirement for high thermal resistance.
5) The wearing comfort and safety should be also considered seriously. The proper materials selection and structure design are essential to comply with the daily requirements of common clothes.

## Conflict of Interest

The authors declare no conflict of interest.
